# Controlling the Guanidinium Cation Rotation by Cation–π Interactions

**DOI:** 10.1002/anie.3934559

**Published:** 2026-05-21

**Authors:** Hannah Busch, Lennart Günzel, Ettore Bartalucci, Robert Schuster, Christian Zocher, Martin Börner, Chandan K. Das, Florian Taube, Björn Corzilius, Maria Fyta, Matthias Ernst, Berthold Kersting, Thomas Wiegand

**Affiliations:** ^1^ Institute of Technical and Macromolecular Chemistry RWTH Aachen University Aachen Germany; ^2^ Max Planck Institute For Chemical Energy Conversion Mülheim a. d. Ruhr Germany; ^3^ Institute of Inorganic Chemistry and Crystallography Leipzig University Leipzig Germany; ^4^ Present address: Laboratory of Computational Chemistry and Biochemistry EPFL Lausanne Switzerland; ^5^ Computational Biotechnology RWTH Aachen University Aachen Germany; ^6^ Center For Computational Life Sciences RWTH Aachen University Aachen Germany; ^7^ Institute of Chemistry and Department of Life, Light & Matter University of Rostock Rostock Germany; ^8^ Institute For Molecular Physical Sciences, ETH Zürich Zürich Switzerland

**Keywords:** cation–π interaction, dynamics, fast magic‐angle spinning, guanidinium, solid‐state NMR

## Abstract

Guanidinium plays an essential role in many disciplines of biology and chemistry, particularly due to its unique role of being engaged in a variety of molecular‐recognition events that are facilitated by the ability to participate in a broad range of noncovalent interactions. Guanidinium cations in salts or perovskite materials are known to rotate along their local symmetry axes rather fast in the order of *ps*, even in the solid state. We herein employ a π‐container to trap a guanidinium cation inside the aromatic cavity by cation–π interactions. X‐ray crystallography and solid‐state nuclear magnetic resonance (NMR) spectroscopy at fast magic‐angle spinning (MAS) frequencies have been utilized to probe the underlying host–guest interactions. The guanidinium motion has been fully characterized by temperature‐dependent MAS‐NMR experiments down to 100 K, as well as by a variety of further solid‐state NMR experiments, and supplemented by quantum‐chemical calculations and molecular dynamics (MD) simulations. Our data point to a restriction of the guanidinium cation rotation about the local C_3_‐axis with correlation times in the order of *ns*. Our study, therefore, showcases that using the calixarene framework as a bearing for trapping a guanidinium cation, we are getting closer to the chemist's dream of controlling molecular rotations.

## Graphical abstract

A lanthanide *π*‐container has been employed to trap a guanidinium cation inside the aromatic cavity by cation–π interactions. X‐ray crystallography and solid‐state nuclear magnetic resonance (NMR) spectroscopy at fast magic‐angle spinning (MAS) frequencies have been utilized to probe the underlying host–guest interactions. Full characterization of the guanidinium cation motion has been achieved by solid‐state NMR, quantum‐chemical calculations, and molecular dynamics (MD) simulations.

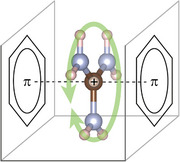

## 1 Introduction

Guanidine, or its protonated form guanidinium, is an essential functional group in biology, where it is, for instance, part of the amino acid arginine or the energy metabolite creatine. Arginine residues play a crucial role in protein folding, as well as nucleotide or nucleic acid binding [1, 2, 3]. Additionally, guanidine derivatives are also of importance in medical drugs like the antidiabetic agent metformin, as a denaturant for proteins, or as antitumor and antimicrobial agents [4, 5]. Various applications of guanidine have also been reported in chemistry, for instance, as a strong organic base (*pK*_a_‐value of 13.5 in water) [6], as a cationic head group of ionic liquids [7], in molecular‐recognition events in supramolecular chemistry [2, 6], in catalysis [8, 9], or as a cation in perovskites. Such perovskites are hybrid organic–inorganic materials with versatile optoelectronic properties often used in solar cells or LEDs, gaining more and more importance as a sustainable energy source [10, 11, 12]. Guanidinium replaces as a monovalent cation the A‐site cation in ABX_3_‐type perovskite crystal structures, substantially improving solar cell performance, such as the optoelectronic properties or stability [13, 14, 15]. These improvements have been linked to the cation dynamics. Rapid rotations along several local rotation axes within the cavities of the perovskite crystal structure have been observed [16]. A detailed solid‐state nuclear magnetic resonance (NMR) spectroscopic study has been reported, in which the type and timescale of the rotation has been thoroughly probed for a variety of cations in single‐ and multi‐cation perovskite compositions, including guanidinium [16].

Albeit the cation dynamics are crucial for the functioning of perovskite materials, the dream of chemists is to control such molecular rotations by designing molecules able to restrict, for instance, the rotation to only a single specific axis. This principle has been explored in the design of a variety of artificial molecular motors [17], in which even unidirectional rotations have been reported [18, 19, 20]. In that vein, noncovalent interactions (NCIs) are attractive for fixing small ligands in larger molecule entities, in which the framework suppresses, for instance, rotations along specific axes due to steric hindrance. Among suitable NCIs, cation–π interactions have gained considerable attraction in the past years due to their occurrence in a variety of chemical and biological contexts (for a comprehensive recent review article see [21]). Examples comprise the binding of alkali cations or cationic organic molecules to aromatic compounds (even in water), the role in stabilizing protein secondary structure, protein–drug interactions, or protein–protein contacts, or the unique role of cation–π interactions in liquid–liquid phase separation processes. In the latter, cation–π interactions between arginine and tyrosine side chains have been identified in driving several phase separation events [22, 23, 24].

**SCHEME 1:**
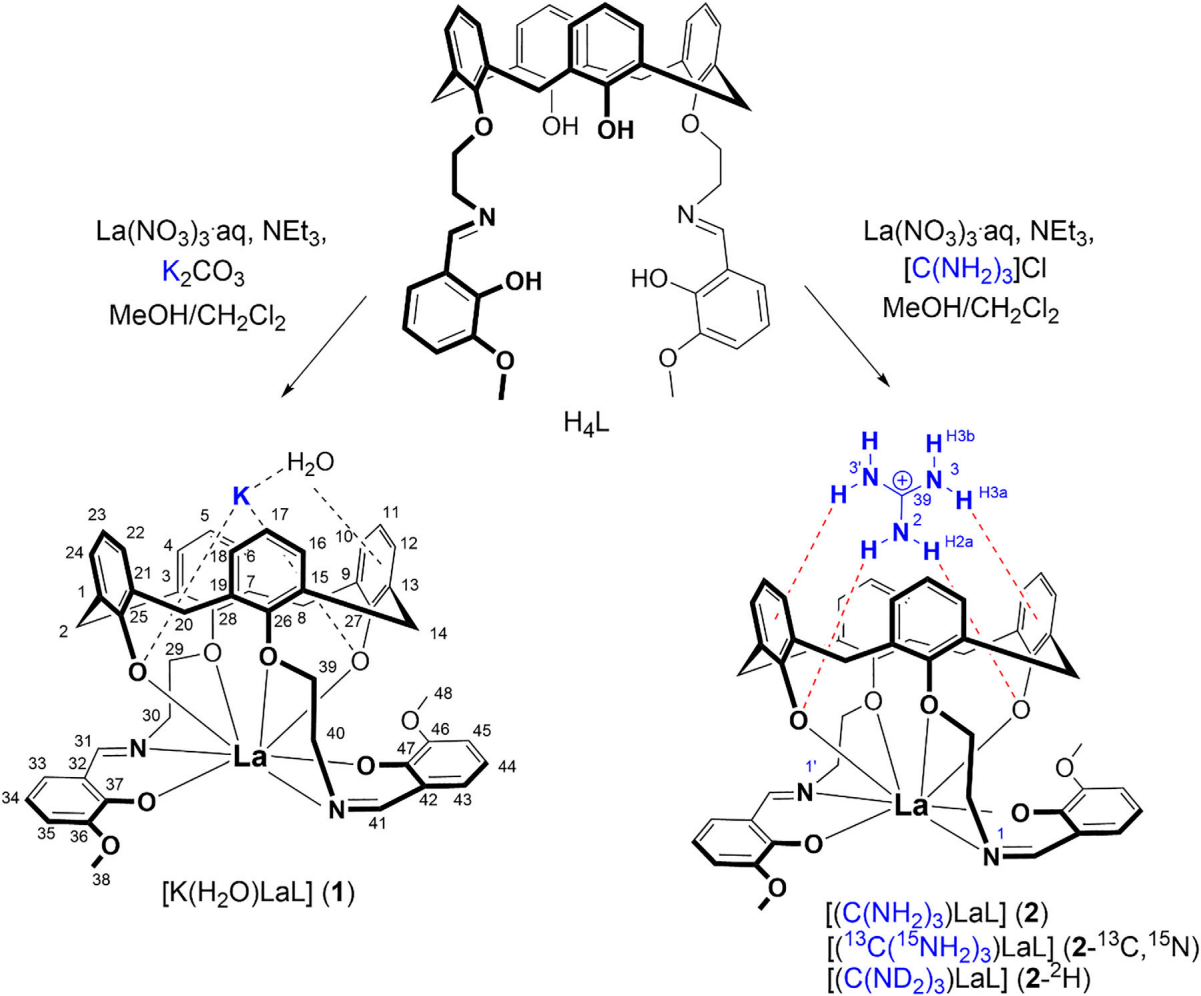
Synthesis and atom labels of compounds described in this study. The labeling scheme used for the C and N atoms of complex **1** was adopted for complex **2**. Additional labels for C, H, and N atoms of the guanidinium ion in **2** are shown in blue.

We herein explore a metalated calix[4]arene system comprising a sandwich‐like πpocket as a bearing for restricting guanidinium cation rotation. Calix[4]arenes have a rich host–guest chemistry [25, 26, 27, 28, 29, 30], and can be utilized for controlling molecular rotations [31, 32]. Solid‐state NMR has been explored among other techniques to probe host–guest interactions in such compounds [25, 26, 28, 33], and to access molecular dynamics of the guest molecules within the cavity [33, 34, 35, 36, 37]. Some of us have recently reported trapping of an isolated K^+^‐H_2_O complex in a π‐pocket of a calix[4]arene lanthanide complex (**1**, Scheme 1) [38]. In this work, we build on our previous study and use the calix[4]arene framework in **2** for stabilizing the guanidinium cation by cation–π interactions, and restricting its rotation along the C_3_‐symmetry axis.

Solid‐state NMR spectroscopy is a powerful tool for both probing the presence and strength of NCIs as well as accessing molecular dynamics with motional time scales ranging from *ps* to *s* [39]. The detection of NCIs by solid‐state NMR has been significantly facilitated in recent years by the increase in accessible magic‐angle spinning (MAS) frequencies (up to 160 kHz these days [40, 41, 42, 43]). This has enabled recording highly‐resolved proton‐detected NMR spectra, closing more and more the gap to solution‐state NMR, where proton‐detection is state‐of‐the‐art for many decades (for seminal work of solid‐state NMR in detecting NCIs even at lower MAS frequencies see [44, 45]). Many applications of proton‐detected solid‐state NMR at MAS frequencies exceeding 100 kHz in both biology and materials sciences have been reported (for some selected review articles, see [46, 47, 48, 49]). The gain in spectral resolution is attributed to a decrease of the so‐called homogeneous contribution to the proton linewidth, which is caused by an insufficient averaging of the proton–proton homonuclear dipolar coupling network at slow‐to‐medium MAS frequencies [50, 51, 52, 53, 54]. The increase in spectral resolution (and lengthening of coherence lifetimes) enables the direct identification of protons engaged in NCIs due to the sensitivity of proton chemical‐shift values on those. In addition, even small ^1^H‐^1^H isotropic *J*‐coupling constants sensitive, for instance, to hydrogen‐bond formation have been observed recently by MAS experiments at 160 kHz [40]. Particularly for samples in which the homogeneous contribution does not dominate the total linewidths, alternatives, such as anti‐z COSY [55], magnetic susceptibility matching [56], or pure isotropic NMR [57] have been reported.

Initially, we focused on the determination of the static structure of complex **2** utilizing x‐ray crystallography, infrared, and proton‐detected NMR experiments at 100 kHz MAS. In the course of these studies, we probed the fascinating dynamic properties of this system employing a variety of solid‐state NMR techniques, namely temperature‐dependent ^15^N MAS‐NMR experiments down to 100 K, relaxation time studies, heteronuclear nuclear overhauser enhancement (NOE), as well as rotational echo double resonance (REDOR) pulse schemes [58]. The experimental data have been complemented by density functional theory (DFT) calculations and molecular dynamics (MD) simulations. This work enabled a thorough investigation and characterization of the guanidinium rotational dynamics in the calix[4]arene environment of complex **2**.

## 2 Results and Discussion

The K^+^/water/calix[4]arene complex **1**, comprising two parallel π‐rings, was considered as a potential host for intercalation of the guanidinium cation in a sandwich‐like fashion. Indeed, simple replacement of K^+^ by a guanidinium cation in the synthetic protocol yielded a pale‐yellow solution from which the targeted complex [(C(NH_2_)_3_)LaL] (**2**) was obtained as an air‐stable solid in 52% yield (Scheme 1). Deuterated and ^13^C,^15^N‐labeled complexes were obtained by analogous reactions in CH_2_Cl_2_/CD_3_OD or utilizing isotopically enriched [^13^C(^15^NH_2_)_3_]Cl, respectively (Section S1).

**FIGURE 1:**
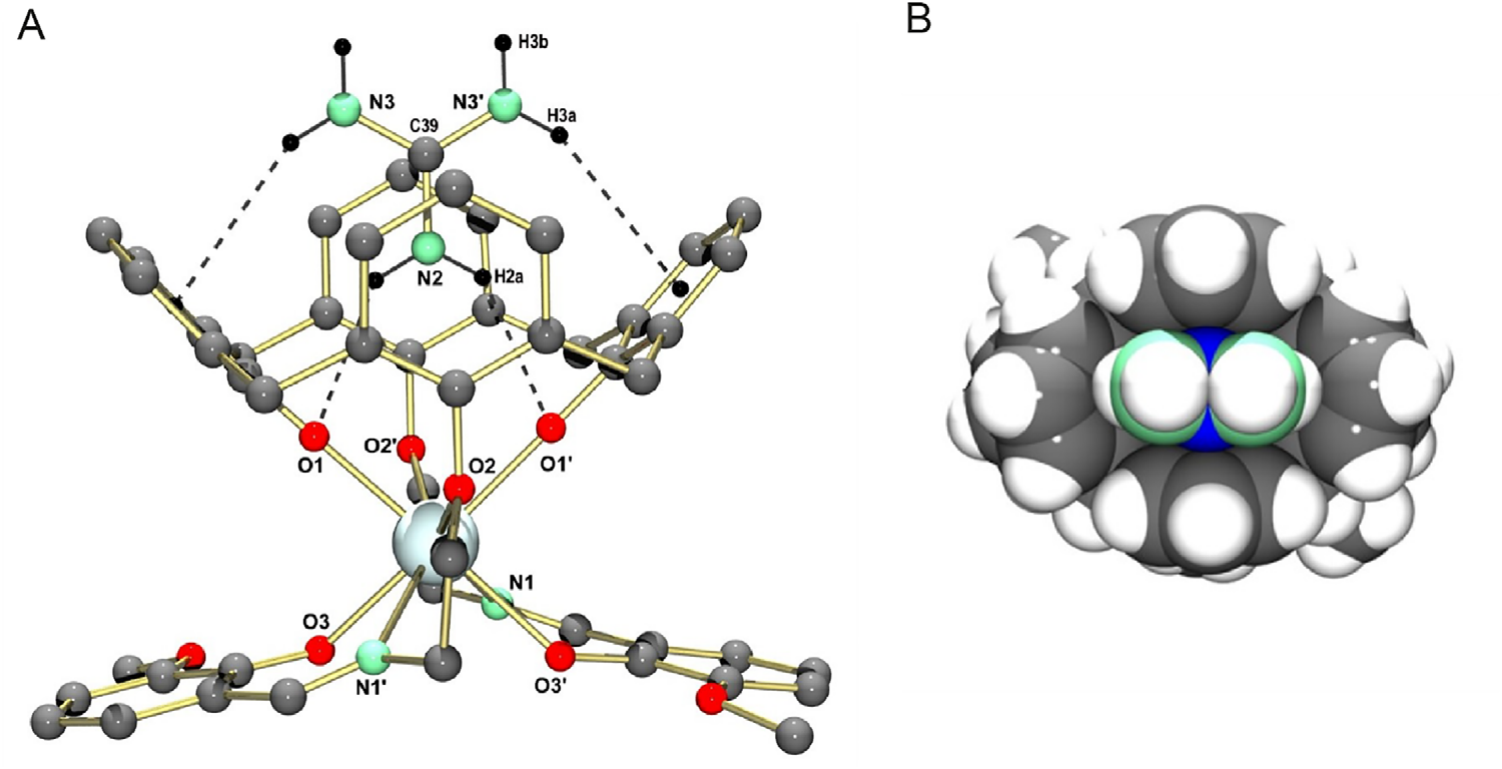
(A) Ball‐and‐stick model of the molecular structure of complex **2** as determined by X‐ray crystallography. Hydrogen atoms of the supporting ligand have been omitted for clarity. Dashed lines represent the intramolecular NH^…^O and NH^…^π bridges (for a plot of the intermolecular NH^…^O hydrogen bonding interactions, see Figure S5). Selected distances / Å: N_2_^…^O1 3.127(4), N_3_^…^centroid(aryl ring) 3.647(3); La‐O1 2.316(2), La‐O2 2.722(2), La‐O3 2.405(2), La‐N1 2.663(3), N_2_^…^La 4.213(3). Symmetry code for equivalent atoms (′): −1 − *x*, 1 + *y*, −1.5 − *z*. (B) Van der Waals plot of complex in **2** (view along the C_2_ axis, top view). Color code: C atom of the guanidinium ion: blue, all other C atoms: dark gray, N: light green, H: white.

The formulation of complex **2** and its isotope‐labeled analogues was unambiguously confirmed by attenuated total reflectance fourier‐transform infrared spectroscopy (ATR‐FTIR) (cf. Section S2, and Figures S1–S4) and X‐ray crystallographic analysis (Figure 1A). Notably, the guest molecule is approximately co‐planar with the two aromatic phenol ether rings, attested by an interplanar angle of 12° between the best planes through the guanidinium ion and the aromatic rings (Figure 1). The distance between the N2 atom and the centroids of the adjacent aryl rings (3.02 Å) is significantly shorter than in other stacked π‐systems. For the benzene dimer, for example, a distance of 3.49 Å has been reported [59]. This indicates the presence of “sandwich‐like” cation–π interactions, as also found in complex **1**. With respect to the dynamic properties presented below, it is worth mentioning that the N_2_^…^O_1_ distance of 3.13 Å is much longer than a typical NH^…^O hydrogen bond. We attribute this to the limited space in the cavity of the calix[4]arene, which hinders closer approach of the guest molecule. The short N^…^centroid(Ar) distances (N3^…^centroid^(aryl)^ = 3.65 Å) suggest that intramolecular NH^…^π interactions with the electron‐rich phenolate rings are present. There are also intermolecular hydrogen bonds between the NH_2_ groups and vanillin O atoms (N3^…^O3 2.84 Å, Figures S5 and S6), which link neighboring complexes of **2**.

### 2.1 Identifying the Guanidinium Cation Resonances by Solid‐state NMR Spectroscopy

**FIGURE 2:**
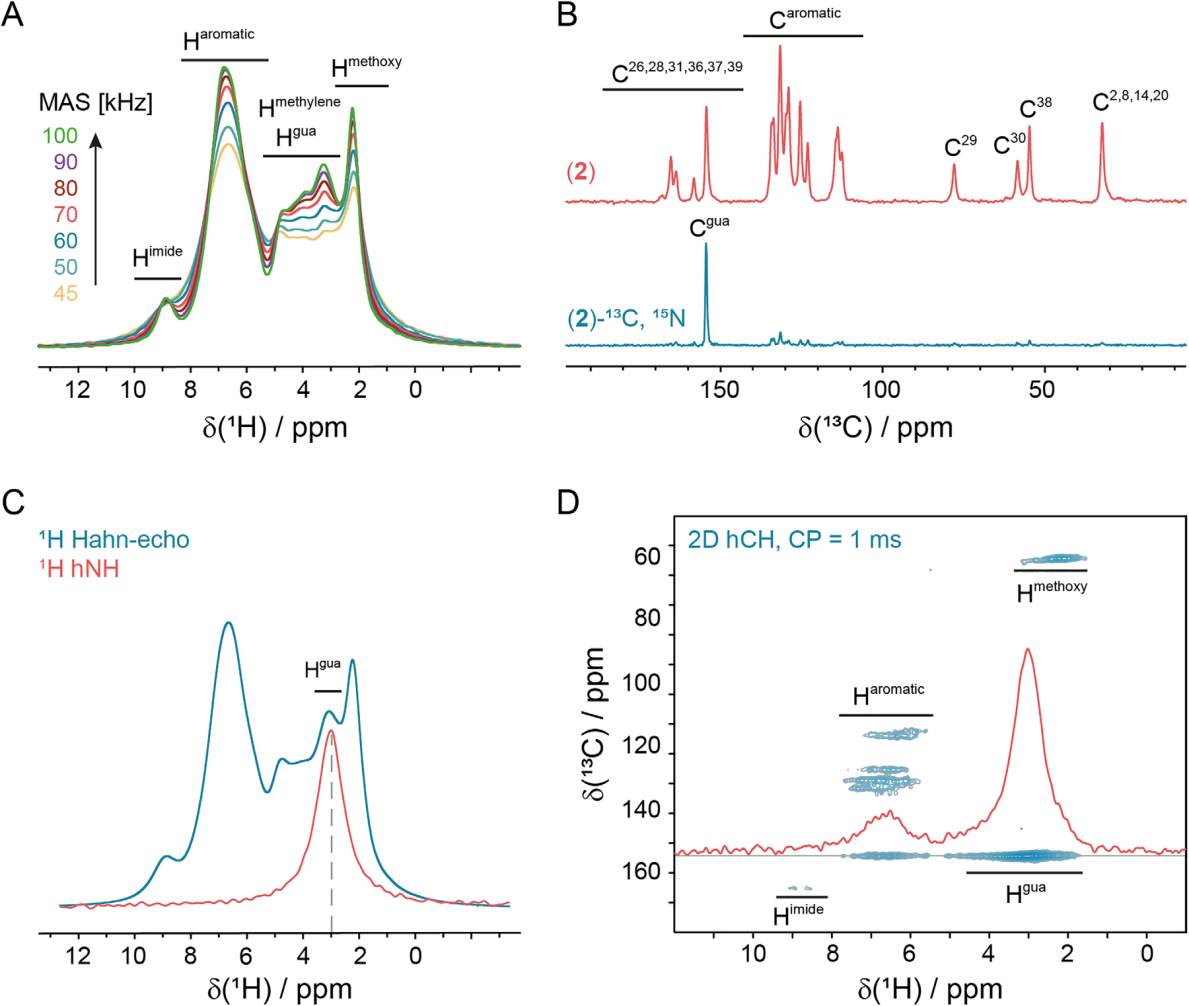
Solid‐state MAS‐NMR characterization of complex **2**. (A) ^1^H MAS‐NMR spectra recorded at different MAS frequencies. The distinguishable spectral regions are marked. (B) Comparison of ^1^H‐^13^C CP‐MAS NMR spectra (60.0 kHz MAS) of the selectively isotope labeled **2**–^13^C,^15^N and unlabeled compound **2**. The assignment of the resonances was transferred from complex **1**. (C) Comparison of a ^1^H‐detected Hahn echo and a 1D hNH spectrum with short contact time spectra (60.0 kHz MAS) of **2**–^13^C,^15^N. (D) 2D hCH spectrum showing proton and carbon correlations of **2**–^13^C,^15^N. At the chemical‐shift value of the guanidinium carbon resonance (154.2 ppm), a slice along F2 is inserted. In all spectra, the distinguishable spectral regions are marked. All spectra were recorded at a magnetic‐field strength of 16.4 T and temperature control (see Section S6.6 and Table S4 for more details on the NMR measurement parameters).

Solid‐state NMR was employed to gain further insight into the structural and dynamic properties of complex **2**. MAS‐dependent ^1^H NMR spectra of **2** with MAS frequencies ranging between 45.0–100.0 kHz were recorded (Figure 2A) [50, 51, 52, 53, 54]. Even at an MAS frequency of 100.0 kHz, the resonances remain rather broad, which points to significant inhomogeneous broadening contributions to the ^1^H MAS linewidths caused by chemical‐shift distributions and/or anisotropic bulk magnetic susceptibility effects [56]. Resonance assignments were transferred from the ^1^H MAS‐NMR spectra of the previously investigated complex **1** [38]. Consistently, the low‐frequency shifted ^1^H resonance at 0.4 ppm that was assigned to the trapped water (for the full details see [38]) in **1** appears to be absent in complex **2**. Figure 2B shows the ^1^H‐^13^C cross‐polarization (CP‐)MAS‐NMR spectrum of **2**. Only a single set of resonances for each carbon site has been observed, in perfect agreement with the C_2_‐symmetry found in the single crystal structure reported above. The unambiguous identification of the ^13^C resonance of the guanidinium cation resonating at 154.2 ppm has been achieved by detecting a ^1^H‐^13^C CP‐MAS NMR spectrum of **2–** ^13^C,^15^N (Figure 2B). The chemical‐shift value is in good agreement with the DFT‐calculated value (155.8 ppm, PBE0/pcSseg‐2) that was obtained on a model of complex **2**, in which only the guanidinium cation within the calixarene cavity has been considered (see Table 1 and Figure S7. For electrostatic potential map calculations using a dimeric model see Figure S8). A similar chemical‐shift value has also been reported for guanidinium sulfate in aqueous H_2_SO_4_ [60, 61]. The guanidinium cation proton resonances (H^2a^, H^3a^, and H^3b^) have been assigned by means of an hNH spectrum in which selectively spatial proximities in the order of a single bond between ^1^H and ^15^N were probed. The hNH spectrum of **2** reveals a single broad resonance at 3.0 ppm, which is thus assigned to the guanidinium protons (Figure S9). In comparison to guanidinium sulfate in a H_2_SO_4_ solution for which a chemical‐shift value of 6.7 ppm has been reported [60], the protons in complex **2** appear to be significantly more shielded, as caused by the ring‐current effects reported for various inclusion complexes using calixarene frameworks. This observation thus supports the presence of a cation–π interaction in complex **2** between the guanidinium ion and the aromatic rings of the calixarene unit.

**TABLE 1:** Isotropic chemical‐shift values, *δ*_iso_, for the nuclei of the guanidinium cation in complex **2**. Experimental values have been obtained from the spectra recorded at an MAS frequency of 60.0 kHz. The DFT‐calculated values (PBE0/pcSseg‐2) are referenced to neat TMS and NH_3_ for ^1^H, ^13^C, and ^15^N, respectively, and were obtained from a simplified model of complex **2** (Figure S7). Geometry optimization of the hydrogen atom positions in the model prior to magnetic‐shielding calculations induces some asymmetry in the model, leading to slightly different chemical‐shift values for the in the crystal structure symmetric atoms (indicated by ′).

Chemical shift δ	^1^H / ppm	^13^C/ ppm	^15^N / ppm
Solid‐state NMR	3.0	154.3	71.1
DFT (PBE0/ pcSseg‐2)	H^2a^/ H^2a′^ = 1.6 / 1.7 H^3a^/ H^3a′^ = 1.3 / 1.4 H^3b^/ H^3b′^ = 2.7 / 2.7 H^mean^ = 1.9	155.8	N^2^ = 98.5 N^3^/ N^3′^ = 85.9

To confirm the encapsulation of the guanidinium cation within the calixarene cavity, a ^13^C‐^1^H hCH correlation spectrum on **2**–^13^C,^15^N was recorded (Figure 2D, for a hCH correlation spectrum on the natural abundance complex used for the resonance assignment see Figure S10). Note that the ^1^H‐^13^C CP transfer turned out to be rather inefficient, pointing to the effect of molecular dynamics (*vide infra*). Indeed, correlations between the ^13^C guanidinium cation resonance at 154.2 ppm and proton resonances at 3.0 and 6.5 ppm were observed, the latter being assigned to aromatic proton resonances of the calixarene ring or the vanillylimine Schiff base units, respectively. As the latter correlation might also be caused by an intramolecular contact with aromatic carbons in natural abundance, a 2D ^1^H‐^1^H spin diffusion (SD)‐based spectrum was recorded, revealing cross peaks between the guanidinium proton resonances and the methoxy protons as well as with aromatic protons, further supporting the observation of spatial proximities between the guanidinium guest and the lanthanide complex host (Figure S11A). We also recorded a 2D ^1^H‐^1^H DQ/SQ correlation spectrum using the Back‐to‐Back (BaBa) recoupling scheme [62, 63]. This spectrum further reveals spatial proximities between the guanidinium proton resonances and the methylene units of the calix[4]arene (Figure S11B).

**FIGURE 3:**
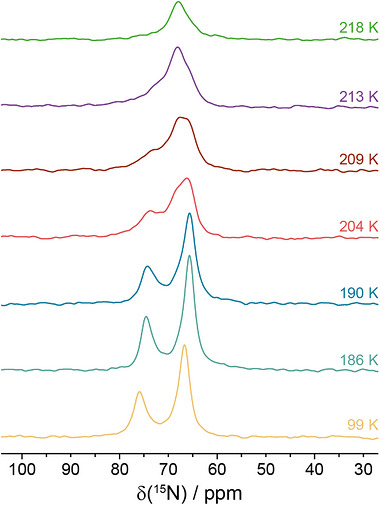
Temperature‐dependent ^1^H‐^15^N CP‐MAS NMR spectra of **2**–^13^C,^15^N recorded at 18.0 kHz MAS and 9.4 T. The coalescence temperature was estimated to be between 213 and 218 K. Note that the spectra were referenced at 100 K and not compensated for any further magnetic‐field drifts upon variation of the temperature.

From DFT calculations of ^1^H chemical‐shift values, however, chemical‐shift differences for the guanidinium proton resonances (up to 1.4 ppm) are expected, which are not observed in the experimental spectra. As the guanidinium proton resonances are still significantly broadened, and therefore the shift difference could be non‐discernible, we turned to ^15^N‐detected MAS spectra. Also, for the nitrogen atoms, two clearly distinguishable ^15^N resonances with a chemical‐shift difference of about 12 ppm were expected based on the DFT calculations. The 1D ^1^H‐^15^N CP‐MAS‐NMR spectrum on **2**–^13^C,^15^N at room temperature (Figure 3), however, reveals only a single resonance for the guanidinium nitrogen sites (N^3^/N^3′^ and N^2^). We hypothesise that molecular motion (such as a rotation along the C_3_‐axis of the guanidinium cation) averages the distinguishable nitrogen positions in the guanidinium cation, which would also explain the single guanidinium proton resonance as outlined above. To verify this hypothesis and further characterize the type and time scale of the motion, additional solid‐state NMR investigations were performed.

### 2.2 Characterization of the Guanidinium Cation Motion by Temperature‐dependent ^15^N Solid‐State NMR Experiments

The motion of the guanidinium cation within the calix[4]arene cavity of complex **2** was initially probed by temperature‐dependent ^1^H‐^15^N CP‐MAS NMR spectra. At the lowest temperature accessible (about 100 K sample temperature), the expected spectrum with two distinguishable ^15^N resonances at 76.0 and 66.8 ppm was observed for the isotope labeled sample **2**–^13^C,^15^N (Figure 3). This corresponds to the slow‐exchange limit, in which the exchange rate constant, *k*_ex_, is significantly smaller than the chemical‐shift difference between the two nitrogen sites. The experimentally observed chemical‐shift difference of 9.2 ppm agrees reasonably well with the DFT‐calculated value (12.5 ppm) obtained from the model of complex **2** as outlined above. The two resonances at 76.0 and 66.8 ppm are assigned to N^2^ and N^3^/N^3’^ based on the expected peak integral ratio of 1:2 as well as the DFT‐calculated values (Table 1). At higher temperatures, the single ^15^N resonance observed thus indicates the fast exchange limit, where *k*_ex_ is much larger than the chemical‐shift difference between the two sites. The averaging of both nitrogen sites points to the already hypothesized rotation of the guanidinium cation about its C_3_‐symmetry axis as discussed in detail further below.

**FIGURE 4:**
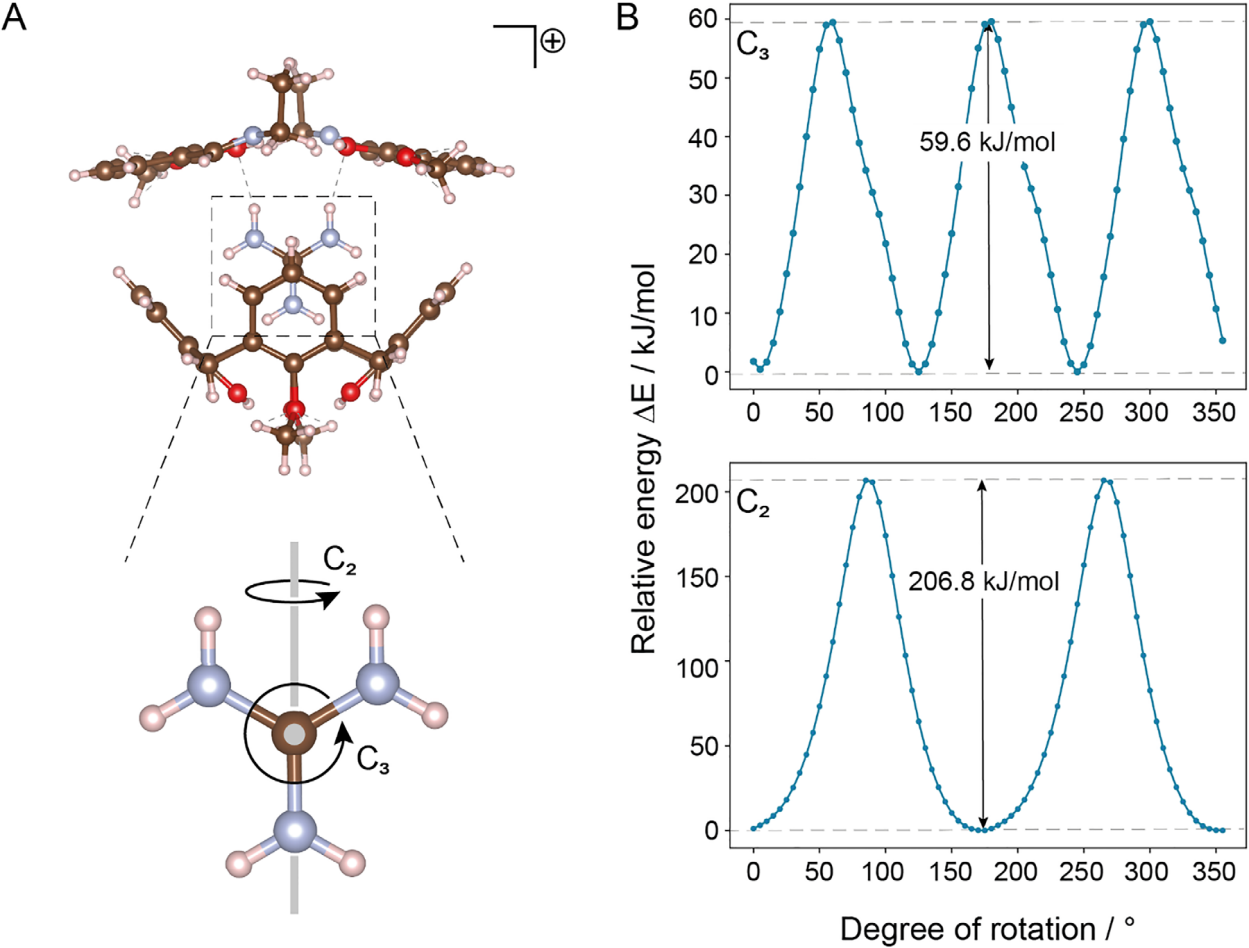
Activation barriers for rotations about the C_2_‐ and C_3_‐symmetry axis in complex **2** calculated by DFT calculations. (A) Simplified model of complex **2** with the local C_3_‐ and C_2_‐rotation axis of the guanidinium cation highlighted. (B) DFT‐calculated (r^2^SCAN‐3c / def2‐mTZVPP) potential energy curves for the guanidinium rotation about the C_3_‐ and C_2_‐axis with the maximum energy difference indicated as activation barrier for the rotation.

From the recorded temperature series (Figure 3), the coalescence temperature (∼215 K) and the exchange rate constant at coalescence (*k*_ex,c_ = 830 ± 20 s^−1^) were estimated. Based on these values, the activation barrier (Gibbs free enthalpy, $\Delta G_{c}^{‡}$) for the rotation was determined to be about 40 ± 3 kJ/mol using the Eyring equation (Section S6.2). The activation energy (*E*_a_) was also calculated by DFT. Rotations along multiple local symmetry axes of the guanidinium cation are *a priori* possible, namely about the local C_3_‐axis and the three C_2_‐axes (see Figure 4A for a schematic depiction). In that vein, the single point energies of the guanidinium cation within the calixarene fragment (for the model see Figure 4A) were computed as a function of the rotation angle around the selected axis. The resulting potential energy surface for the C_3_‐rotation (Figure 4B) shows three minima corresponding to rotations of 0°, 120°, and 240° and three maxima corresponding to rotations of 60°, 180°, and 300°. The activation barrier is calculated to around 60 kJ/mol. Deviations to the experimental value might be explained by the absence of thermostatistical corrections in such calculations, as well as by the simplified model used. Additionally, the potential energy curve for a C_2_‐rotation about the C^39^─N^2^ bond aligning with the C_2_‐axis of the complex was calculated, from which an activation barrier of about 200 kJ/mol has been determined (Figure 4B). The high energy barrier arises since the calixarene moiety poses a steric hindrance for this rotation and we conclude that this rotation does not occur under the studied experimental conditions.

### 2.3 Probing the Time Scale of Guanidinium Cation Rotation

**FIGURE 5:**
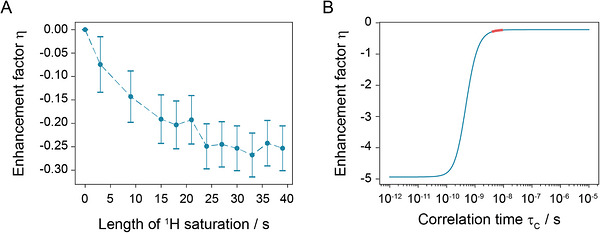
HetNOE experiments of complex **2**–^13^C,^15^N. (A) Enhancement factor *η* for the guanidinium cation ^15^N resonance determined as a function of the length of the proton saturation. For proton saturation times > 25 *s*, an equilibrium state is reached. The error bars are based on the signal‐to‐noise ratios of single spectra (for details see Supporting Information). (B) Enhancement factor for the {^1^H}^15^N hetNOE of an NH_2_ group dependent on the correlation time τ_c_ of the motion. The experimentally determined enhancement factor (including its uncertainty) of complex **2** is marked in red.

In order to probe the time scale of the guanidinium cation rotation, we next turned to a variety of solid‐state NMR experiments, namely heteronuclear Nuclear Overhauser Effect (hetNOE), ^13^C and ^15^N longitudinal relaxation times, and ^13^C‐^15^N REDOR experiments. HetNOE effects are well established in solution‐state NMR, but have been less often observed in the solid state, since the necessary fast local molecular motions often do not exist in solids. Nevertheless, several examples in the solid state have been reported [64, 65, 66]. Herein, the ^15^N{^1^H} hetNOE for complex **2–** ^13^C,^15^N was probed at room temperature by saturating the proton transitions (steady‐state NOE) [67]. The enhancement factor *η* is defined by the ratio of the integral of the unsaturated resonance, *I*_0_, and the integral at different proton saturation times, *I*(*t*), as $\eta \left(t\right)=\frac{I\left(t\right)\;-\;I_{0}}{I_{0}}$ (see Figure S12 for an exemplary saturated and unsaturated ^15^N spectrum). In case of **2**, the steady‐state value of *η* is determined to −0.25 ± 0.02 (Figure 5A), leading to a correlation time (*τ_c_*) in the range of 10^−9^ *s* (for the analytically‐calculated *η*‐values as a function of *τ_c_*, assuming that the relaxation is purely dipolar and using Lorentzian spectral density functions [67, 68] see Figure 5B). Note that the NH_2_ system can be treated equivalently to an NH system in the absence of significant cross‐correlated cross‐relaxation and assuming that the protons relax faster than the nitrogen, as reported previously [67, 69]. The hetNOE turns out to be not sensitive enough to characterize the motional correlation time of the guanidinium cation precisely, but the order of magnitude for *τ_c_* can be estimated.

We thus determined ^13^C and ^15^N longitudinal relaxation times (*T*_1_) for the guanidinium cation resonances at four temperatures ranging from 260 to 290 K at 60.0 kHz MAS (Table 2). To deduce the correlation time *τ*_c_ from the *T*_1_ relaxation times, a stochastic three‐site jump model with equal probabilities within the model‐free framework [39, 70, 71] was employed based on the geometry and distances of the guanidinium cation found in the single‐crystal structure. Assuming that the relaxation is primarily of a dipolar nature and neglecting any cross‐relaxation pathways, the guanidinium ^13^C relaxation rate in complex **2** is given by:

$$\begin{array}{c} R_{1,\;13C}=6{\left(\frac{\delta_{CH}}{4}\right)}^{2}\cdot \left[J\left(\omega_{H}-\omega_{c}\right)+3J\left(\omega_{c}\right)+6J\left(\omega_{H}+\omega_{c}\right)\right]\\ +3{\left(\frac{\delta_{CN}}{4}\right)}^{2}\cdot \left[J\left(\omega_{N}-\omega_{c}\right)+3J\left(\omega_{c}\right)+6J\left(\omega_{N}+\omega_{c}\right)\right]. \end{array}$$

**TABLE 2:** Measured longitudinal relaxation times *T_1_* for the guanidinium ^13^C and ^15^N resonances of complex **2** at different temperatures. The error on the *T_1_* relaxation times was estimated from the exponential fitting of experimental curves. Based on a three‐site jump model, the correlation time *τ_c_* for the C_3_‐rotation of the guanidinium was determined from the *T*_1_ relaxation times.

Temperature / K	*T_1_* ^13^C/ s	*τ_c_* ^13^C / ns	*T_1_* ^15^N / s	*τ_c_* ^15^N / ns
260	382 ± 19	460	42 ± 4	1150
270	323 ± 17	380	35 ± 3	980
280	255 ± 11	300	23 ± 1	630
290	191 ± 11	230	18 ± 1	490

Here, δ_*CH*_ and δ_*CN*_ are the dipolar coupling constants of ^13^C with ^1^H and ^15^N, respectively, with (γ_I_ and γ_S_ represent the gyromagnetic ratios):

$$\;\delta_{IS}=-2\frac{\mu_{0}}{4\pi }\frac{\gamma_{I}\gamma_{S}\hbar }{{r_{IS}}^{3}},$$

and *J*(ω) are the spectral density functions as a function of either the ^1^H, ^13^C, or ^15^N nuclear Larmor frequencies (*ω*_H_, *ω*_C_, or *ω*_N_) for the three‐site jump model under MAS with the correlation time *τ*_c_ and the order parameter *S^2^* given by:

$$J\;\left(\omega \right)=\frac{2}{5}\;\left(1-S^{2}\right)\cdot \frac{\tau_{c}}{1+{\left(\tau_{c}\omega \right)}^{2}}.$$

**FIGURE 6:**
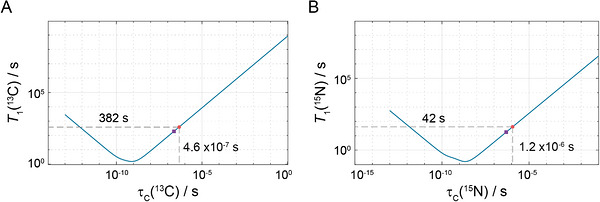
Longitudinal relaxation times enable the determination of correlation times *τ*_c_. The relaxation times for ^13^C (A) and ^15^N (B) were analytically calculated by assuming a three‐site jump model as a function of the correlation time of the C_3_‐rotation. The measured values for *T_1_* and the corresponding results for *τ_c_* at 260 K are labelled by red dots and dashed lines. The additional purple squares show the values for 290 K.

For the order parameter, a value of $S=-\frac{1}{2}$ was used. The guanidinium ^15^N resonance was treated equivalently, taking only dipolar relaxation from the two directly bound protons into account. As the *T*_1_ relaxation times of both ^13^C and ^15^N decrease at higher temperatures, the slow‐motion regime of the calculated curves (a motion slower than the Larmor frequency) needs to be evaluated. From the calculated curves (Figure 6), the correlation time *τ_c_* for the C_3_‐rotation at the lowest temperature was determined to be about 460 and 1150 ns from the ^13^C and the ^15^N *T*_1_ relaxation times, respectively. The difference between the two correlation times can be attributed to additional relaxation pathways or additional motional modes that are not considered in this simple model. Such pathways include auto‐ and cross‐correlated contributions to auto‐ and cross‐relaxation rate constants or additional motions like 180° jumps of the NH_2_ groups or vibrational motions around the equilibrium position.

Finally, the guanidinium rotation was characterized by a ^13^C‐^15^N REDOR experiment, which is typically used to measure the heteronuclear dipolar‐coupling constant (and thus internuclear distances) under MAS [39, 58]. In case of molecular motion, the REDOR curves are affected by a corresponding modulation of the heteronuclear dipolar‐coupling constant [72]. And indeed, the experimental REDOR curve of **2** shows strong deviations from the rigid limit case using the distances found in the crystal structure, as expected in the case of the rotation about the C_3_‐axis (Section S6.4 and Figure S13).

### 2.4 Wobbling Motion About the C_3_‐Axis Studied by ^13^C Chemical‐Shift Anisotropy Analysis

The last question we tried to address is whether the guanidinium cation in complex **2** undergoes a tilting motion about the C_3_‐axis by random fluctuation (like a “wobbling”). Therefore, a slow‐spinning ^13^C MAS‐NMR spectrum was recorded, which allows determining the chemical‐shift anisotropy (CSA) of the guanidinium ^13^C atom (Figure S14A). The fitting of the ^13^C line shape indicated an axially symmetric CSA tensor (Table S3), as expected for the C_3_‐rotation discussed above (Figure S14B,C). In case of an additional “wobbling” motion, also $\delta_{\mathrm{zz}}^{\mathrm{PAS}}$, which points along the C_3_‐rotation axis, should decrease in comparison to the rigid limit. DFT calculations of the ^13^C magnetic‐shielding tensor mimic the static case. A comparison of the experimental and DFT‐calculated $\delta_{\mathrm{zz}}^{\mathrm{PAS}}$ components reveals rather similar values ($\delta_{\mathrm{zz},\exp}^{\mathrm{PAS}}$ = 68 ppm and $\delta_{\mathrm{zz},\mathrm{DFT}}^{\mathrm{PAS}}$ = 53 ppm) in light of uncertainties in the experimental line shape fitting and in the model used for DFT. Our data thus allow to hypothesize that the wobbling motion along the C_3_‐axis is either absent or occurs only about a very small opening angle. ^2^H MAS investigations do not reveal further insights into the molecular motion (Section S6.6 and Figure S15).

### 2.5 MD Simulations Support the Experimental Observations

**FIGURE 7:**
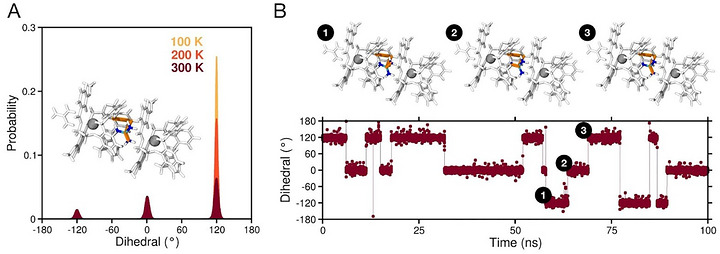
(A) Probability distribution of the dihedral angle corresponding to the C_3_ rotation of the guanidinium rotor at three different temperatures, obtained from five independent 100 ns long MD simulations at each temperature. The C_3_ rotational dihedral angle is highlighted in orange. The corresponding time trace is shown in the Supporting Information (Figure S16). (B) Representative time trace of the C_3_ rotational dihedral angle from a 300 K simulation, including MD snapshots corresponding to the labelled regions in the dihedral dynamics. These illustrate random, jump‐like rotations of +120° or −120°. A 100 ns MD simulation video further demonstrates the stochastic rotary motion of the guanidinium rotor at 300 K; see Video S1.

Further confirmation for the restriction of the motion to a C_3_‐rotation is obtained by all‐atom MD simulation. The simulation was performed with the dimeric form of complex **2** (Figure 7) in the gas phase at three different temperatures: 100, 200, and 300 K, respectively. For each temperature, five independent MD trajectories each of 100 ns duration were generated within the canonical (NVT) ensemble, resulting in a cumulative simulation time of 1.5 µs. To probe the C_3_‐rotation of the guanidinium ion within the complex, the dihedral angle between one of the C─N bonds of the guanidinium and the connecting straight line between two para‐positioned carbon atoms of one phenolate of the calixarene moiety was monitored throughout the simulation (Figure 7A). Notably, the probability distribution of the dihedral angle associated with C_3_‐rotation at the three temperatures shows that no rotational motion was observed at 100 and 200 K. This implies that the thermal energy at these temperatures is not sufficient to overcome the rotational energy barrier of the guanidinium ion within complex **2**, as also observed in the low‐temperature ^15^N MAS‐NMR spectra. In contrast, at 300 K, spontaneous rotation of the guanidinium ion around its C_3_‐axis was observed. The time evolution of the same dihedral angle from a representative MD trajectory (Figure 7B) further reveals that the C_3_‐rotational motion of the guanidinium ion within complex **2** occurs in a jump‐like, stochastic fashion, characterized by rapid ‐on the scale of roughly tens of *ns*‐ +120° or −120° rotational transitions interrupted by dwell times in corresponding symmetry‐equivalent energy minima. However, consistent with the DFT calculation results, no rotation of the guanidinium ion around its C_2_‐axis was observed in the MD simulations.

## 3 Conclusion

The calixarene cavity of a lanthanide complex has been employed herein to (i) trap a guanidinium cation by a cation‐π interaction and (ii) use this interaction as a bearing for controlling the rotation of the guanidinium cation. ATR‐FTIR, X‐ray crystallography, and solid‐state MAS‐NMR confirmed that the guanidinium cation indeed engages in a network of NCIs, comprising a cation–π interaction as well as multiple hydrogen bonds, the latter though being only transient at room temperature due to the guanidinium cation rotation. Calixarene moieties provide a versatile framework for such cation–π interactions due to their electron‐rich, preorganized cavities of tunable sizes [73]. In case of complex **2**, the cation–π interaction between the positively charged guanidinium cation and the electron‐rich π‐system of the calixarene is facilitated by the nearly coplanar alignment of the guanidinium cation and two aromatic phenol ether moieties. The observed strongly shielded guanidinium cation proton ^1^H NMR chemical‐shift value accounts for the shielding effect caused by aromatic ring‐current effects in a cation–π interaction, as reported, for example, for an asparagine residue in a peptide [74].

Guanidinium cation dynamics in the solid state have been reported in several studies, including various guanidinium salts such as halides [75], nitrates [76], and perchlorates [77], as well as a mixed methylammonium–guanidinium perovskite [16]. These investigations employed solid‐state NMR techniques, including NMR relaxometry, to analyze the types and timescales of the rotational motion. In most cases, not only a rotation about the guanidinium's C_3_‐axis has been reported, but also motions around the C_2_‐axes were observed. The activation barriers for C_3_‐rotation in various crystalline guanidinium salts range from 8.5 to 85 kJ/mol [78], which are of similar magnitude to the determined Gibb's free energy ΔG^‡^ of approximately 41 kJ/mol, as well as the DFT calculated activation energy of about 60 kJ/mol found for complex **2**. However, in contrast to the fast motional time scales in the picoseconds regime observed for the hexachloroantimonate salt [78] or the guanidinium perovskite at room temperature [16], significantly slower motion in the range of hundreds of nanoseconds was observed for compound **2**. This difference may be attributed to the transient formation of hydrogen bonds between the guanidinium cation and the calixarene unit of the complex (Figure 1). In addition, solid‐state NMR and MD simulations indicate that the rotation of the guanidinium cation in **2** is mostly restricted to the C_3_‐rotation, in contrast to the examples mentioned above.

Controlling molecular rotations or the conformation of a molecule by the restriction of a rotation is a powerful approach for modulating materials’ properties, as well as for directing chemical reactions. In photochemistry, for instance, this principle is used when the previously hindered rotation of a double bond becomes possible upon light irradiation, leading to isomerization of a compound. If the rotation is hindered due to the viscosity of a solvent, molecules such as the fluorescent rotary dye DPTPA4 serve as a viscometer [79]. In luminescent molecules in general, suppressing rotations can reduce nonradiative deactivation pathways and therefore enhance emission properties [80]. Furthermore, in the field of nanotechnology and artificial molecular machines, the control over rotation is essential for designing molecular motors and subsequently using those for catalysis, smart materials, or self‐assembling systems, amongst others [17, 81]. In several studies, even controlling the sense of rotation (unidirectional rotations) in such molecular rotors has been reported [19].

In conclusion, complex **2** highlights the possibility of the calixarene moiety to engage in cation–π interaction for trapping small molecules in the cavity. The NCIs between the guanidinium cation and the host, along with the steric hindrance of the cavity, effectively suppress any other molecular motion than the guanidinium cation C_3_‐rotation as evidenced by combining solid‐state NMR spectroscopy, quantum‐chemical calculations, and classical MD all‐atom simulations. Our study contributes to the dream of chemists in controlling molecular rotations by developing advanced synthetic protocols. The trapping of the guanidinium moiety opens the way for incorporating further biological compounds, such as arginine and lysine residues, in the calixarene cavity.

## Author Contributions

L.G. and R.S. synthesized the samples. H.B. and E.B. recorded the solid‐state NMR spectra, analysed those and performed the DFT calculations. H.B. and F.T. performed the low‐temperature NMR experiments. C.Z. created the NCI plots. M.B. determined the single‐crystal structure. C.K.D. carried out the MD simulations. M.E. wrote the script for analysing the relaxation data and simulated the REDOR curves in GAMMA. B.K. and T.W. designed the research, which was supervised by B.C., M.F., B.K. and T.W. All authors contributed to the writing of the manuscript.

## Conflicts of Interest

The authors declare no conflicts of interest

## Supporting information




**Supporting File**: The authors have cited additional references within the Supporting Information [82, 83, 84, 85, 86, 87, 88, 89, 90, 91, 92, 93, 94, 95, 96, 97, 98, 99, 100, 101, 102, 103, 104, 105, 106, 107, 108, 109, 110, 111, 112, 113, 114, 115, 116, 117, 118, 119, 120, 121, 122, 123, 124, 125, 126, 127, 128].

## Data Availability

The data supporting this article has been included as part of the ESI. Crystallographic data for compound **2** have been deposited at the CCDC under CCDC‐2500293. All NMR raw data are deposited under RADAR4Chem (doi: 10.22000/ytfddayjzw0nx4p5).
