# Supplementary material for: Controlling the Guanidinium Cation Rotation by Cation–π Interactions
Source: Angew Chem Int Ed Engl. 2026 May 21;65(29):e3934559. doi: 10.1002/anie.3934559 (PMC13360552; doi:10.1002/anie.3934559)
Supplement: Supplementary file 1 — Supporting File: The authors have cited additional references within the Supporting Information [82, 83, 84, 85, 86, 87, 88, 89, 90, 91, 92, 93, 94, 95, 96, 97, 98, 99, 100, 101, 102, 103, 104, 105, 106, 107, 108, 109, 110, 111, 112, 113, 114, 115, 116, 117, 118, 119, 120, 121, 122, 123, 124, 125, 126, 127, 128]. [file ANIE-65-e3934559-s001.docx]

**Supporting Information**

**Controlling the Guanidinium Cation Rotation by Cation-π Interactions**

Hannah Busch^a,b,‡^, Lennart Günzel^c,‡^, Ettore Bartalucci^a,b,d^, Robert Schuster^c^, Christian Zocher^c^, Martin Börner^c^, Chandan K. Das^e,f^, Florian Taube^g^, Björn Corzilius^g^, Maria Fyta^e,f^, Matthias Ernst^h^, Berthold Kersting^c,*^, and Thomas Wiegand^a,b,*^

^a^ Institute of Technical and Macromolecular Chemistry, RWTH Aachen University, Worringerweg 2, 52074 Aachen, Germany

^b^ Max Planck Institute for Chemical Energy Conversion, Stiftstr. 34-36, 45470 Mülheim/Ruhr, Germany

^c^ Institute of Inorganic Chemistry and Crystallography, Leipzig University, Johannisallee 29, 04103 Leipzig, Germany

^d^ present address: Laboratory of Computational Chemistry and Biochemistry, EPFL Lausanne, Av. François-Alphonse Forel 3, 1015 Lausanne, Switzerland

^e^ Computational Biotechnology, RWTH Aachen University, Worringerweg 3, 52074 Aachen, Germany

^f^ Institute of Chemistry and Department of Life, Light & Matter, University of Rostock, Albert-Einstein-Straße 2, 18059 Rostock, Germany

^g^ Institute for Molecular Physical Sciences, ETH Zürich, Vladimir-Prelog-Weg 2, 8093 Zürich, Switzerland

^‡^: Both authors contributed equally.

^*^: Corresponding authors: B.K. b.kersting@uni-leipzig.de, T.W. thomas.wiegand@cec.mpg.de

**Table of content**

1. Experimental section S3
2. ATR-FTIR procedure and spectra S8
3. X-ray crystallography S12
4. Analysis of electron density function S14
5. DFT calculations S15
6. Additional NMR data and experimental details S16
   1. Additional solution state NMR spectra S16
   2. Spectra of natural abundance complex **2** (solid-state NMR) S17
   3. Calculation of the Gibbs free energy S18
   4. Nuclear Overhauser Effect: exemplary spectra (solid-state NMR) S18
   5. REDOR data (solid-state NMR) S19
   6. Guanidinium ^13^C chemical-shift anisotropy (solid-state NMR) S21
   7. Deuterium NMR (solid-state NMR) S22
   8. Experimental data (solid-state NMR) S23
7. Molecular Dynamics Simulations S27
8. Supplementary references S28

**Experimental Section**

The vanillylimine-functionalized calix[4]arene ligand (abbreviated as H_4_L) was obtained according to a literature procedure^[1]^. All reagents and solvents were commercial grade and used without further purification. Elemental analyses were done on a Vario EL elemental analyzer from Elementar Analysensysteme GmbH, Hanau. Fourier-transform infrared spectra were recorded at room temperature on a Bruker VERTEX 80v FT-IR spectrometer utilizing a platinum ATR unit (1 cm^-1^ resolution). The diffuse reflectance spectra were collected at room temperature on a Jasco V-670 UV-vis-NIR spectrophotometer with an ARN-914 absolute reflectance measurement unit, equipped with a photomultiplier and a PbS photoconductive cell. The complexes were mixed with BaSO_4_ powder in a mortar to a content of 5 % (by weight). The spectrum was collected in the 190 – 1400 nm range with a resolution of 1 nm. The FTIR spectra were plotted with ORIGINPRO 2019 v9.6.0.172 from ORIGINLAB CORPORATION^[2]^. Non-covalent interactions plots (NCI plots) were obtained using the NCIPLOT software package and are based on promolecular densities^[3-4]^. The promolecular densities were calculated from the sum of the atomic contributions, taking into account the respective atom positions. Real-space images were generated using ChimeraX v1.8 and Gnuplot v5.4.8^[5-7]^.

**Synthesis and Analysis of Compounds. Synthesis of [(C(NH_2_)_3_)LaL]** (**2**). A solution of H_4_L (50 mg, 64 mmol) in 15 mL CH_2_Cl_2_ was treated with NEt_3_ (40.3 mmol, 289 mmol, 4.00 eq). To this mixture, a solution of La(NO_3_)_3_·6H_2_O (34.3 mg, 77 mmol, 1.20 eq) in 10 mL CH_3_OH was added, followed by the subsequent introduction of a solution of guanidinium chloride (8.0 mg, 83 mmol, 1.30 eq) in 5 mL CH_3_OH. Crystallization occurred over the course of 16 h, affording well-formed crystals in the absence of stirring. The crystals were isolated by filtration, washed thoroughly with CH_2_Cl_2_ and CH_3_OH, and dried at 60 °C to constant weight. The product was obtained as pale-yellow green crystals (32 mg, 51 % based on H_4_L). UV-vis (solid compound, 5% in BaSO_4_): *λ_max_* (nm) = 195, 204, 232, 273, 307, 370, 593. ATR-IR: $\tilde{\nu}$ (cm^-1^) = 3450 (w), 3429 (w), 3050 (w), 2913 (w), 2880 (w), 2843 (w), 1660 (s), 1626 (s), 1593 (s), 1546 (m), 1469 (s), 1443 (s), 1388 (s), 1346 (m), 1241 (s), 1217 (s), 1191 (s), 1160 (m), 1078 (s), 1040 (s), 964 (m), 928 (w), 852 (s), 835 (m), 764 (m), 734 (s), 675 (m). Elemental analysis for C_49_H_48_LaN_5_O_8_∙2H_2_O (973.8+36.0): calc. C 58.28, H 5.19, N 6.93 %; found C 58.30, H 4.81, N 6.83 %. This compound was additionally characterized by X-ray crystallography. Crystals of **2** suitable for X-ray crystallography were picked from the reaction mixture.

**Synthesis of [(^13^C(^15^NH_2_)_3_)LaL] (2-^13^C,^15^N).** The isotopically labeled analogue [(^13^C(^15^NH_2_)_3_)LaL] (**2-**^13^C,^15^N) was prepared in analogy to **2** using isotopically enriched guanidinium chloride [^13^C(^15^NH_2_)_3_]Cl (98 atom % ^15^N, 99 atom % ^13^C) under otherwise identical conditions. This compound was identified by ATR-FTIR spectroscopy (ν_as_(ND_2_) 2592, 2565 cm^-1^; ν_s_(ND_2_) 2458 cm^-1^) (cf. Supplementary Section 2) and NMR spectroscopy.

**Synthesis of [(C(ND_2_)_3_)LaL] (2-^2^H).** The deuterated derivative [(C(N^2^H_2_)_3_)LaL] (**2-**^2^H) was prepared in analogy to **2** using CH_3_OD instead of CH_3_OH. This compound was identified by ATR-FTIR spectroscopy (ν_as_(ND_2_) 2592, 2565 cm^-1^; ν_s_(ND_2_) 2458 cm^-1^) (cf. Supplementary Section 2) and NMR spectroscopy.

**X-ray crystallography.** A suitable single crystal of **2** was selected and mounted on a cryoloop using perfluoropolyether oil. The data set was collected at 180(2) K on a Stoe Stadivari X-ray diffractometer equipped with a GeniX 3D Cu-HF (Xenocx) microfocus X-ray source with a graded multilayer mirror (Cu-K_α_, λ = 1.54186 Å) and a hybrid pixel detector Pilatus3 300K (Dectris). Data processing was carried out with the Stoe X-Area software including a spherical absorption correction and scaling routine. The structures were solved with SHELXT 2018/2^[8]^ using dual methods and refined by full-matrix least-squares techniques based on all data against F^2^ using version 2018/3 SHELXL^[8]^ and Olex2^[9]^. The coordinates of all non-hydrogen atoms were refined with anisotropic thermal parameters. Hydrogen atoms were included on idealized positions except for the guanidinium guest. The H-atoms bonded to the guanidinium have been located in the final Fourier map and were refined using the 1.2-fold *U_iso_* value of the parent atom (see also Supplementary Section 3).

**Crystallographic Data for [(C(NH_2_)_3_)LaL],** C_49_H_48_LaN_5_O_8_, *M*_r_ = 973,83 monoclinic, space group *C*2*/c*, *a* = 20.6029(5) Å, *b* = 10.5230(2) Å, *c* = 21.3312(4) Å, *β* = 111.255(2)°, *V* = 4310.11(16) Å^3^ , *Z* = 4, *ρ*_calc_ = 1.501 g/cm^3^, *T* = 180(2) K, *μ*(Cu-K*_α_*) = 8.170 mm^-1^ (*λ* = 1.54186 Å), crystal size 0.23 × 0.20 × 0.16 mm^3^, 14293 reflections measured, 4080 unique, 3969 with *I* > 2*σ*(*I*). Final *R*_1_ = 0.0272 (*I* > 2*σ*(*I*)), *wR*_2_ = 0.0752 (all data), 296 parameters and 0 restraints, min./max. residual electron density = −0.369/0.727 e/Å^3^.

**Density function theory calculations.** Density functional theory (DFT) calculations for a model of complex **2** (see Figure S7) were performed with the implementation in ORCA, versions 5.0.1^[10]^ or 6.1.0^[11]^. The magnetic shielding tensors of the nuclei-of-interest were calculated by first performing a constraint geometry optimization starting from the crystal structure and using the r^2^SCAN-3c composite density functional and a modified triple-ζ basis set (def2-mTZVPP)^[12]^. The resolution-of-identity approximation (RI) was employed with a def2/J auxiliary basis set^[13]^ and Grimme’s dispersion correction was performed (D4)^[14]^ as well as a geometric counterpoise correction (gCP)^[15]^. In the optimization, the positions of all atoms except for hydrogen were fixed, as the heavy atoms are already accurately defined by the crystal structure. The magnetic-shielding tensors were calculated with the Gauge-Including Atomic Orbital (GIAO) approach employing the PBE0 hybrid density functional^[16]^ and triple-ζ pcSseg-2 basis set^[17]^. For Coulomb- and exchange-type integrals, the resolution-of-identity approximation (RI-JK) was employed with automatically generated auxiliary basis sets^[18]^. A gCP correction was performed as well. For determining the chemical-shift values from the calculated isotropic magnetic-shielding values, the shielding of tetramethyl silane (TMS) and ammonia (NH_3_) were calculated on the same level-of-theory, the first was used for ^1^H and ^13^C, the latter for ^15^N.

The rotation energy surfaces were determined by calculating the single point energies (SPE) for the different rotamers of the guanidinium in a simplified dimer fragment (Figure 4A). Here, the same level of theory as in the previous geometry optimization was employed (r^2^SCAN-3c and def2-mTZVPP)^[12]^. An explicit Counterpoise correction via the Boys and Bernardi procedure^[19]^ for the Basis Set Superposition Error (BSSE) was calculated once for the rotamers of 0° and 60°. Since these SPE values showed only minimal differences to the gCP-corrected SPE values (in the order of 1 kJ/mol for the energy difference of the rotamers), no subsequent explicit corrections were performed for all of the rotamers.

**MD Simulation.** All-atom molecular dynamics (MD) simulations of the dimeric form of complex **2** (see Figure 7) were performed using the GROMACS 2024.4^[20]^ software package. The initial structure of complex **2** was obtained from the X-ray crystal structure reported in this work. The general AMBER force field (GAFF)^[21]^ was employed to describe the organic calixarene framework and the guanidinium rotor, with restrained electrostatic potential (RESP)-derived partial charges assigned to all atoms. The Lennard-Jones parameters for the La ions were adopted from the work of Li *et al*^[22]^. The coordination of La was modelled through non-bonded interactions (Lennard-Jones and Coulombic), without defining any explicit bonded potential term for the La-ligand coordination bonds. All simulations were conducted under periodic boundary conditions using a simulation box of dimensions 12.6 x 13.9 x 12.8 nm³. The simulations were performed under gas-phase conditions, i.e., without explicit or implicit solvent. In order to emulate the solid-state crystal packing environment, a harmonic position restraint with a force constant of 10.000 kJ mol⁻¹ nm⁻² was applied to all atoms of the dimer (see Figure 7) except the guanidinium ion (levitating between two La ions), which was left unrestrained to allow free rotational motion inside the dimer structure. These restraints were maintained throughout the simulations to preserve the structural integrity of the dimer complex. MD simulations were carried out at three different temperatures: 100 K, 200 K, and 300 K.

Prior to production runs, the system underwent energy minimization, followed by a heating phase from 0 K to the target temperature over 200 ps in the canonical (NVT) ensemble. Subsequently, 100 ns production MD simulations were carried out under the canonical ensemble. All bonds involving hydrogen atoms were constrained using the LINCS^[23]^ algorithm, enabling the use of a 2 fs time step with the leap-frog integrator. The system temperature was maintained at the target temperature using the velocity-rescaling thermostat^[24]^ with a coupling constant of 0.1 ps. Short-range non-bonded interactions were truncated at 1.0 nm, while long-range electrostatic interactions were treated using the particle-mesh Ewald (PME)^[25]^ method. For each temperature (100 K, 200 K, and 300 K), five independent simulations (each of a 100 ns duration) were initiated with distinct random seeds to generate different initial velocity distributions. In total, these simulations yielded a cumulative trajectory length of 1.5 µs, which was used for statistical analysis of the rotational dynamics of the guanidinium ion.

In order to ensure the absence of rotation around the C_2_ axis, we performed an additional set of three independent 100 ns long MD simulations (in the (NVT) ensemble at 300 K) during which a positional restraint was applied to one of the C–N bonds of the guanidinium ion, while the other two C–N bonds were allowed to move freely. This setup would, in principle, only allow rotation of the guanidinium ion around the C_2_ axis passing through the restrained C–N bond. None of these simulations revealed a C_2_ rotational motion.

**Solid-state NMR spectroscopy.** Solid-state NMR spectra were recorded in 0.7 mm or 1.3 mm Bruker triple-resonance MAS probes on a 16.4 T Bruker AVANCE NEO spectrometer. Experiments were performed at 60.0 kHz or 100.0 kHz MAS frequency in case of the 1.3 mm and the 0.7 mm probe, respectively. The ^13^C chemical-shift anisotropy was determined from slow-spinning MAS spectra (11.0 kHz MAS).

All experiments at 60.0 kHz MAS were recorded with temperature control with a nominal temperature of 270-290 K and an air gas flow of 300-600 L/h (for experimental details see Supplementary Section 6.6, Supplementary Table S4). The ^1^H and ^13^C chemical-shift values were referenced to TMS and ^15^N chemical-shift values to liquid NH_3_. In all cases, adamantane was used as a secondary standard^[26]^. Radiofrequency pulses were optimized directly on the sample using radio frequency powers of 100 to 150 kHz on ^1^H, 100 kHz on ^13^C and 83.3 kHz on ^15^N. For the adiabatic cross-polarization experiments, a tangential ramp^[27-28]^ was employed to match the Hartman-Hahn condition. Proton decoupling was achieved by WALTZ64 decoupling with an ^1^H rf-frequency of 5 kHz^[29]^.

The recorded spectra were processed with the software Topspin (4.1.4 and 4.4.0). For 1D spectra an exponential-decaying (LB_max_ = 50 Hz) and for 2D spectra a sifted sine-bell apodization function (SSB = 2) were used. Zero-filling was applied up to double the amount of the recorded points, and a baseline correction was performed if necessary.

*Low temperature ^15^N MAS experiments*

Low temperature ^15^N-detected spectra were performed on a Bruker AVANCE III HD spectrometer with a Bruker ASCEND DNP 9.4 T wide bore magnet and a 1.3 mm Bruker triple-resonance LTMAS DNP probe. The MAS frequency was set to 18.0 kHz. The temperature has been calibrated using an external reference sample of KBr following the procedure of Thurber and Tycko^[30]^ which allowed to directly correlate the sample temperature within the MAS NMR rotor under the same settings of bearing, drive and variable temperature (VT) gas flow and temperature settings. The spectra were referenced to liquid NH_3_ using methionine as a secondary standard^[31]^. A linear ramp was used in the CP polarization transfer step.

*Heteronuclear Nuclear Overhauser Effect (hetNOE)*

The hetNOE was measured using a published pulse sequence based on ^1^H saturation prior to ^15^N-detection^[32]^ with an additional saturation comb on ^15^N before the recycle delay and the ^1^H saturation pulses. Peak fitting was performed with ssNake (v1.4)^[33]^ and the enhancement factor and correlation time were determined as described in ^[34]^. The error on the enhancement factor was estimated by the signal-to-noise ratios of the spectra and transferred to the correlation time by using inverse-variance weighting.

**2. ATR-FTIR**

Figure S1 shows the Attenuated Total Reflectance Fourier-Transform Infrared Spectroscopy (ATR-FTIR) spectrum of microcrystalline **2** in the range of 3800 to
3000 cm^-1^. Two bands with different intensities at 3432 and 3338 cm^-1^ are identified, which can be assigned to the asymmetric (ν_a_) and symmetric (ν_s_) NH_2_ stretching vibrations, respectively, of the guanidinium cation. Both bands are significantly redshifted (Δν_redshift_ 22-33 cm^-1^) and clearly split (shoulders at 3446 cm^-1^, 3363 cm^-1^, Δν_splitting_ = 14 and 25 cm^-1^) when compared with those observed in [C(NH_2_)_3_][ClO_4_] (ν_a_ 3454, ν_s_ 3371 cm^-1^)^[35]^. We attribute the observed redshifts in **2** to intra- and intermolecular NH^…^O hydrogen bonds between the guanidinium cation and the phenolate O atoms of the La complex. The splitting of the IR bands can be attributed to a lowering of the symmetry of the guanidinium cation from the ideal *D*_3h_ symmetry (free guanidinium ion) to a local *C*_2v_ symmetry in the La complex (as also observed in the crystal structure described further below). The deuterated and ^13^C,^15^N-labeled samples (**2**-^2^H and **2**-^13^C,^15^N) were also characterized by ATR-FTIR spectroscopy, which confirms the assignments (see Figures S2-S4 and Table S1). In **2**-^2^H, all the ν(NH) bands disappear, and new bands for the corresponding ND modes are detected at 2592 and 2565 cm^-1^ (ν_as_(ND_2_)) and 2458 cm^-1^ (ν_s_(ND_2_), respectively (Table 1, Figure S4). The wavenumber ratio ν(NH)/ν(ND) for each pair of corresponding NH/ND stretches in **2** and **2**-^2^H is in the range 1.33-1.36, in good agreement with the theoretical value of 1.37. Similarly, for **2**-^13^C^15^N all the ν(NH) bands are shifted to lower wavelengths by up to 20 cm^-1^, again confirming the assignments (Figures S2 and S3).

**Table S1.** Selected IR data for compounds **2**, **2**-^2^H and **2**-^13^C,^15^N

| **Compound** | **ν_as_(NH_2_) / cm^-1 [a]^** | **ν_s_(NH_2_) / cm^-1 [a]^** |
| --- | --- | --- |
| **2**, [(C(NH_2_)_3_)LaL] | 3446 (sh), 3432 | 3360, 3338 |
| **2-D**, [(C(ND_2_)_3_)LaL] | 2592, 2565 | 2458 (br) |
| **2-^13^C^15^N**, [(C(ND_2_)_3_)LaL] | 3437 (sh), 3418 | 3352 (sh), 3319 |

^[a]^ Band maxima were obtained by spectral deconvolution. For the FTIR spectra of compounds **2**-^2^H and **2**-^13^C,^15^N see Figures S1-S4.


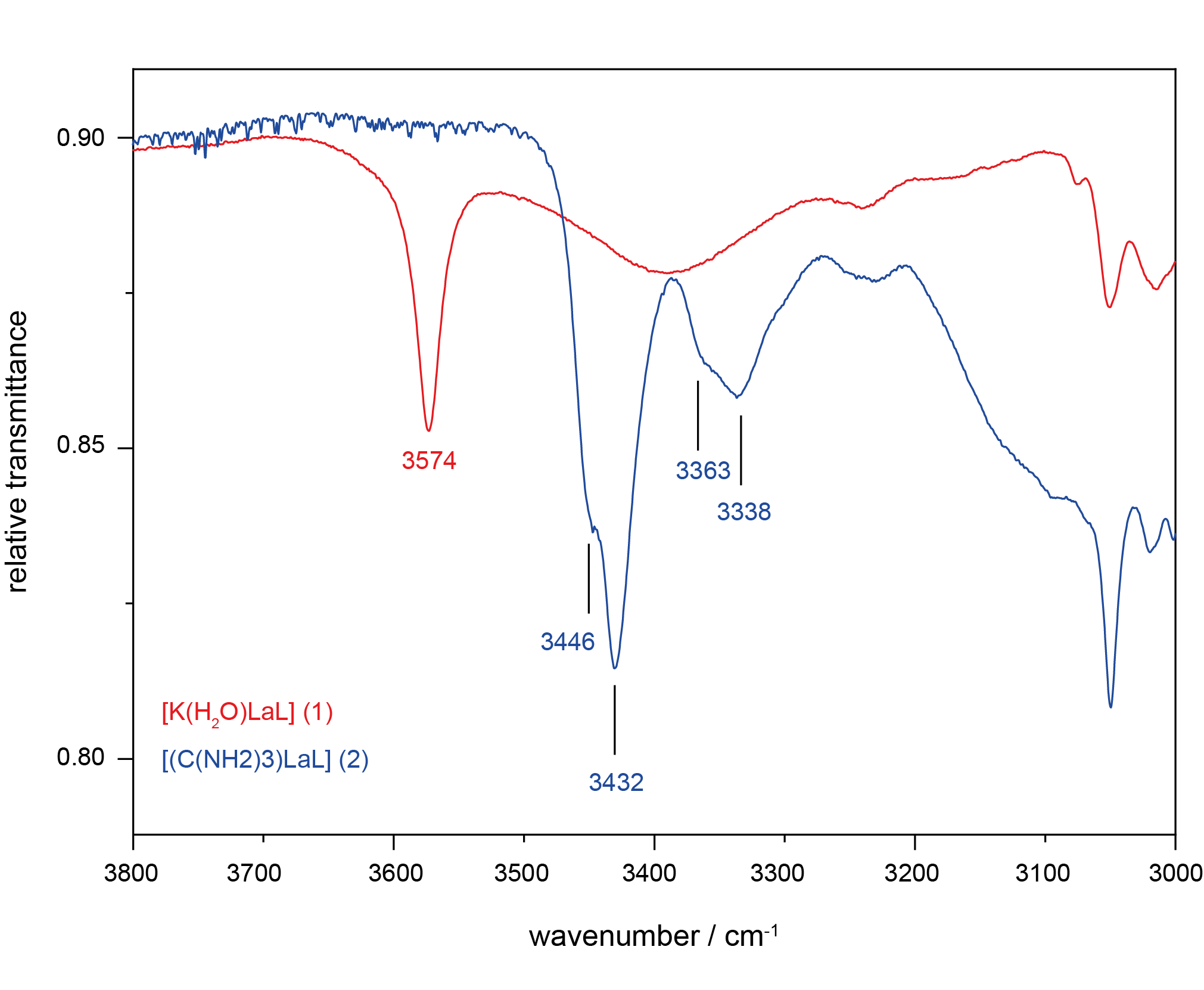


**Figure S1.** ATR-FTIR spectrum of **2** at 295 K. The numbers refer to the stretching frequencies of the band maxima (or shoulders) of the NH stretching vibrations. The ATR-FTIR spectrum of **1** is shown for comparative purposes (taken from reference ^[36]^).


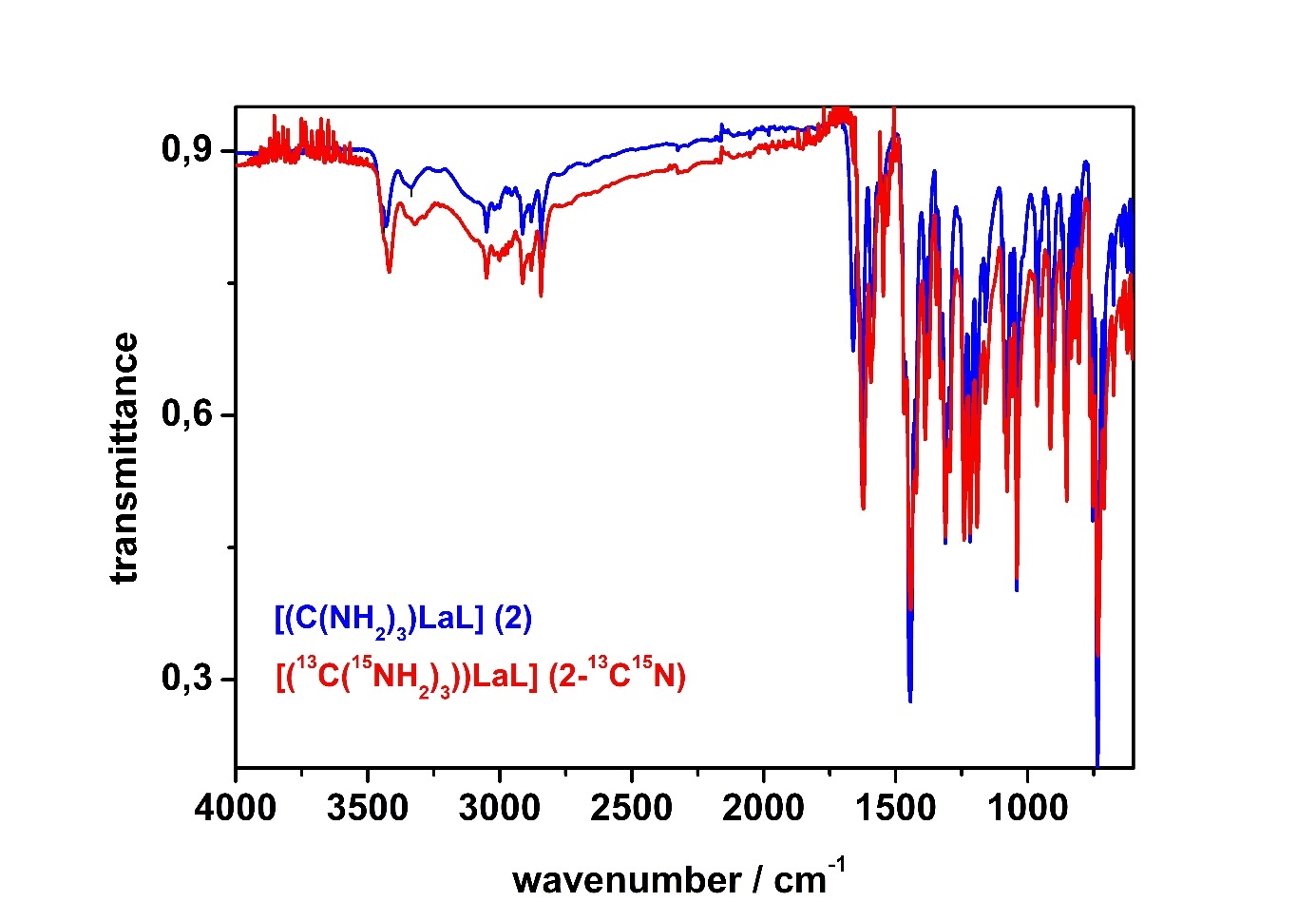


**Figure S2.** ATR-FTIR spectrum of **2-**^13^C,^15^N at 295 K from 4000- 600 cm^-1^. The spectrum for **2** is shown for comparison.


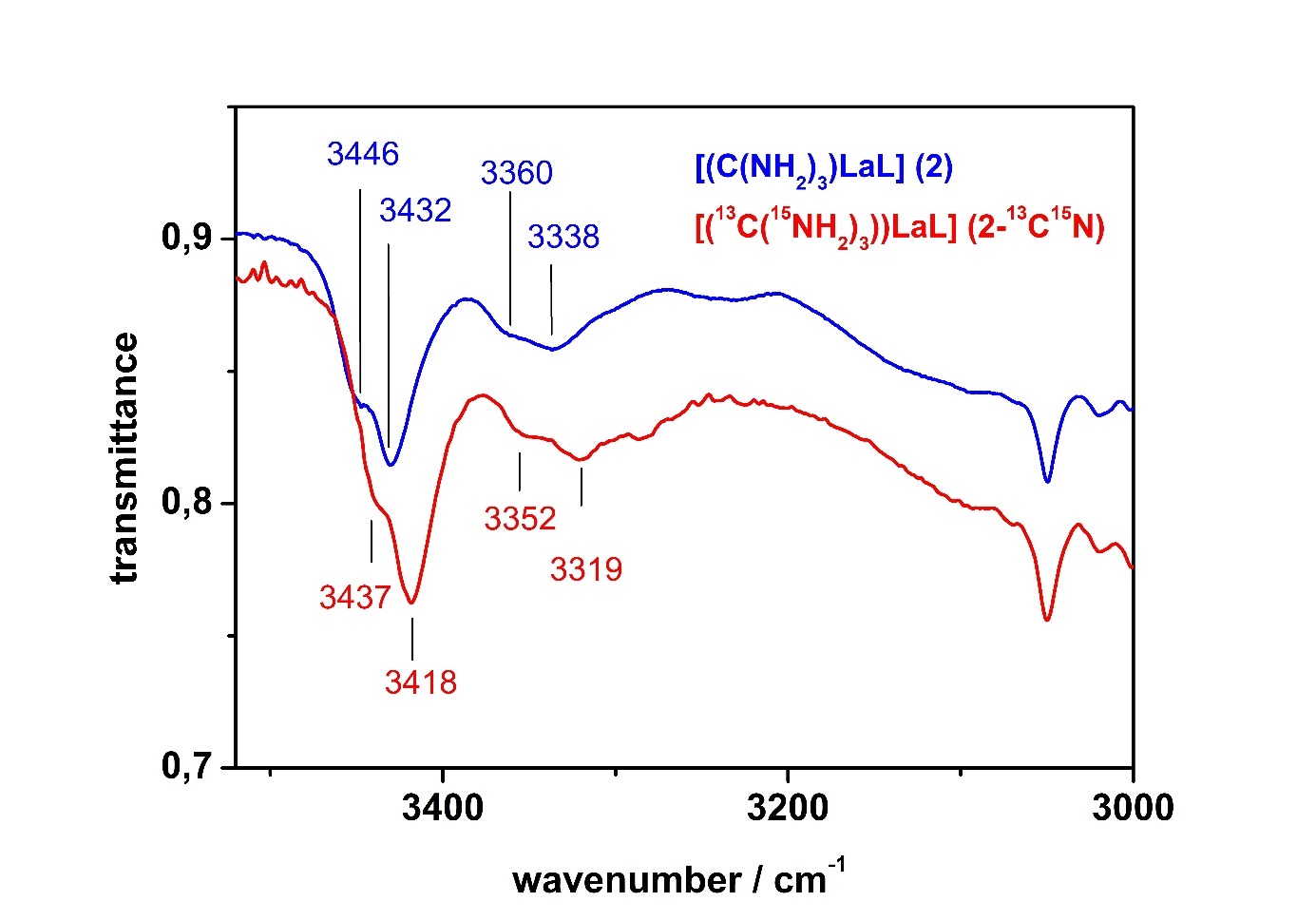


**Figure S3.** ATR-FTIR spectrum of **2-**^13^C,^15^N at 295 K from 3650-3000 cm^-1^. The spectrum for **2** is shown for comparison. The numbers refer to the shoulders (or maxima) of the NH stretching vibrations.


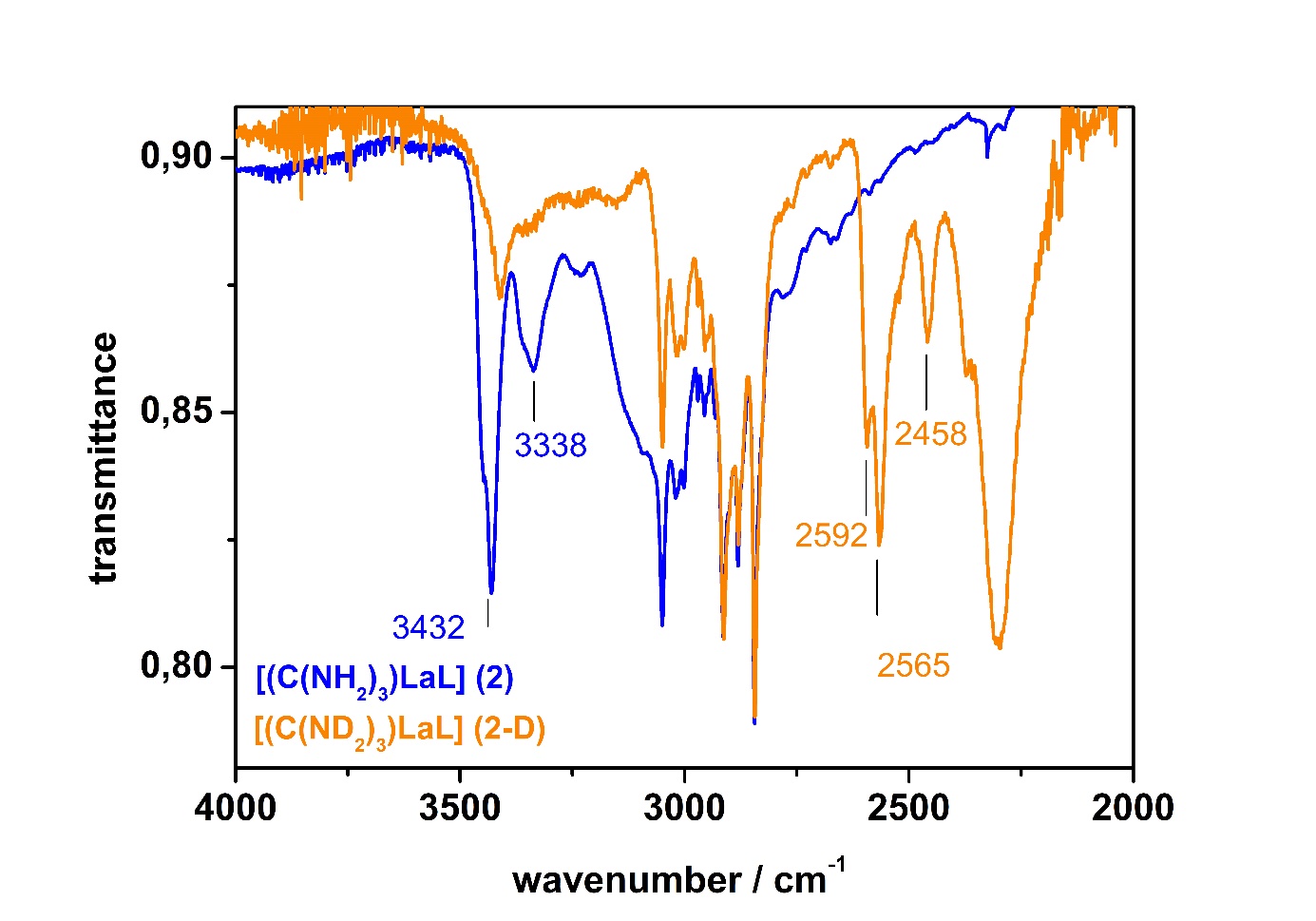


**Figure S4.** ATR-FTIR spectrum of **2-**^2^D at 295 K (from 4000-2000 cm^-1^). The spectrum for **2** is shown for comparison The numbers refer to the shoulders (or maxima) of the NH (for **2**) or ND stretching vibrations (for **2-D**).

**3. X-ray crystallography**


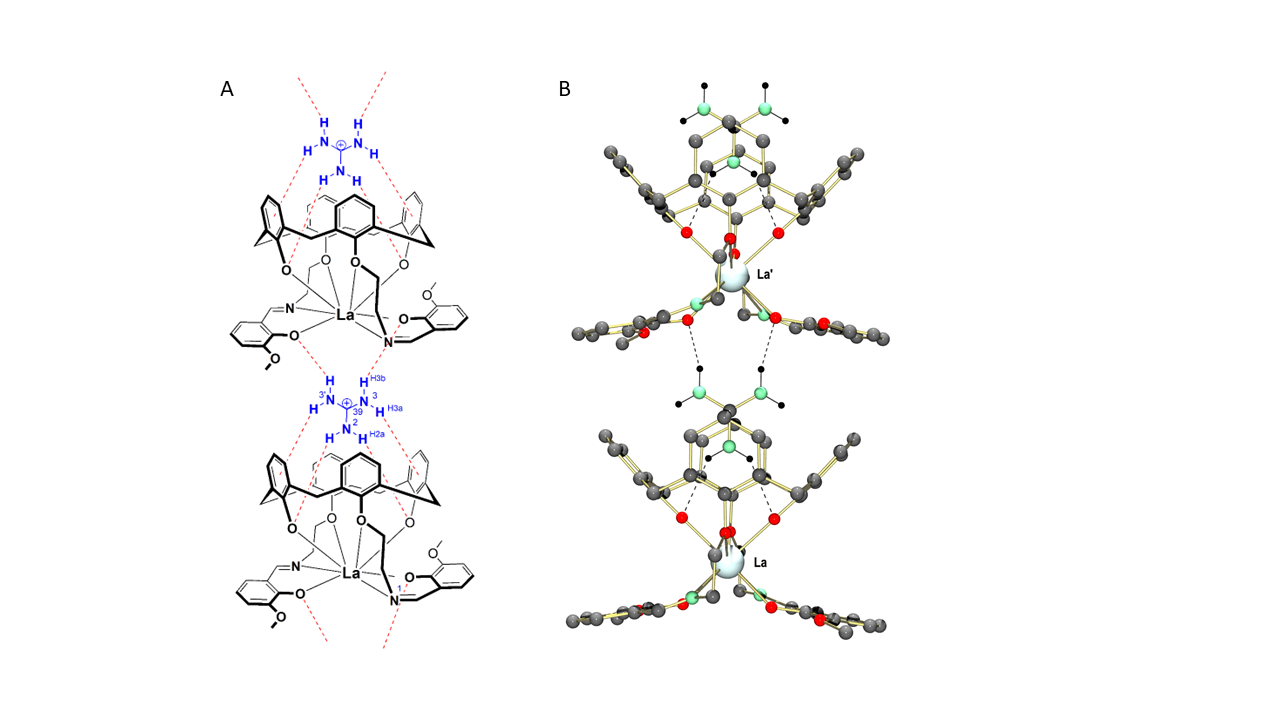


**Figure S5**. Intermolecular hydrogen bonding interactions in **2** derived from the X-ray crystallographic structure (A: schematic view, B: ball-and-stick model generated with ORTEP 3 or windows^[37]^ and POVRAY^[38]^).

**Determination of Coordination Geometries utilizing SHAPE**

The coordination geometries of the lanthanide complex was examined utilizing the SHAPE program^[39]^. Crystallographic coordinates were converted to orthogonal coordinates. According to SHAPE, deviations from ideal coordination geometry are represented by a symmetry factor that increases upon increasing distortions from the ideal geometry, for which the deviation factor is zero.

According to SHAPE, the coordination geometry of the eight-coordinate La^3+^ ion in [(C(NH_2_)_3_)LaL] (**2**) is best described as as distorted, doubly capped trigonal prismatic^[39]^. However, the differences between this coordination geometry to some others (such as triangular dodecahedral, or snub disphenoidal (J84)) are not very pronounced (see the grey highlighted areas in Supplementary Table S2). The La-O and La-N distances vary largely between 2.32 and 2.72 Å. The twist angles between the La1O1O1′ and La1O3O3′ planes and the La1O2O2′ and LaN1N1′ planes are 21.5° and 30.1°, respectively. The intramolecular La^…^N2 distance in **2** is 4.213 Å, which is slightly shorter than the corresponding La^…^K^+^ distance in **1** (4.347 Å)^[36]^.

**Table S2**. SHAPE symmetry factors of the [(C(NH_2_)_3_)LaL]-complex (**2**).

| **Coordination Geometry** | **Abbreviation** | **Symmetry** | **2** |
| --- | --- | --- | --- |
|  |  |  |  |
| Octagon | OP-8 | D_8h_ | 30.966 |
| Heptagonal pyramid | HPY-8 | C_7v_ | 23.401 |
| Hexagonal bipyramid | HBPY-8 | D_6h_ | 9.883 |
| Cube | CU-8 | O_h_ | 6.321 |
| Square antiprism | SAPR-8 | D_4d_ | 4.798 |
| Triangular dodecahedron | TDD-8 | D_2d_ | 4.276 |
| Johnson - Gyrobifastigium J26 | JGBF-8 | D_2d_ | 9.349 |
| Johnson - Elongated triangular bipyramid (J14) | JETBPY-8 | D_3h_ | 22.458 |
| Johnson - Biaugmented trigonal prism (J50) | JBTP-8 | C_2v_ | 3.668 |
| Biaugmented trigonal prism | BTPR-8 | C_2v_ | 4.170 |
| Snub disphenoid (J84) | JSD-8 | D_2d_ | 3.620 |
| Triakis tetrahedron | TT-8 | T_d_ | 6.950 |
| Elongated trigonal bipyramid | ETBPY-8 | D_3h_ | 20.744 |

**4. Analysis of the electron density function**

A topological analysis of the electron density function (*ρ*(r)) for complex **2** according to Bader’s *Atoms in Molecules* theory was performed to shed some light on the nature of the noncovalent interactions^[40-41]^. Figure S6 shows the AIM/NCI plots for the inter- and intramolecular NH^…^O and NH^…^π bonding interaction between the guanidinium ion and the calix[4]arene fragment. As can be seen, both NCI plots reveal attractive NH^...^O and NH^…^ π interaction as clearly indicated by a negative value of sign(λ_2_) ρ(r) shown in blue or green. As expected, the NH^…^O bonds are much stronger than the NH^…^π interactions with negative sign(λ_2_) ρ(r) values ranging from -0.03 to -0.02 a.u..


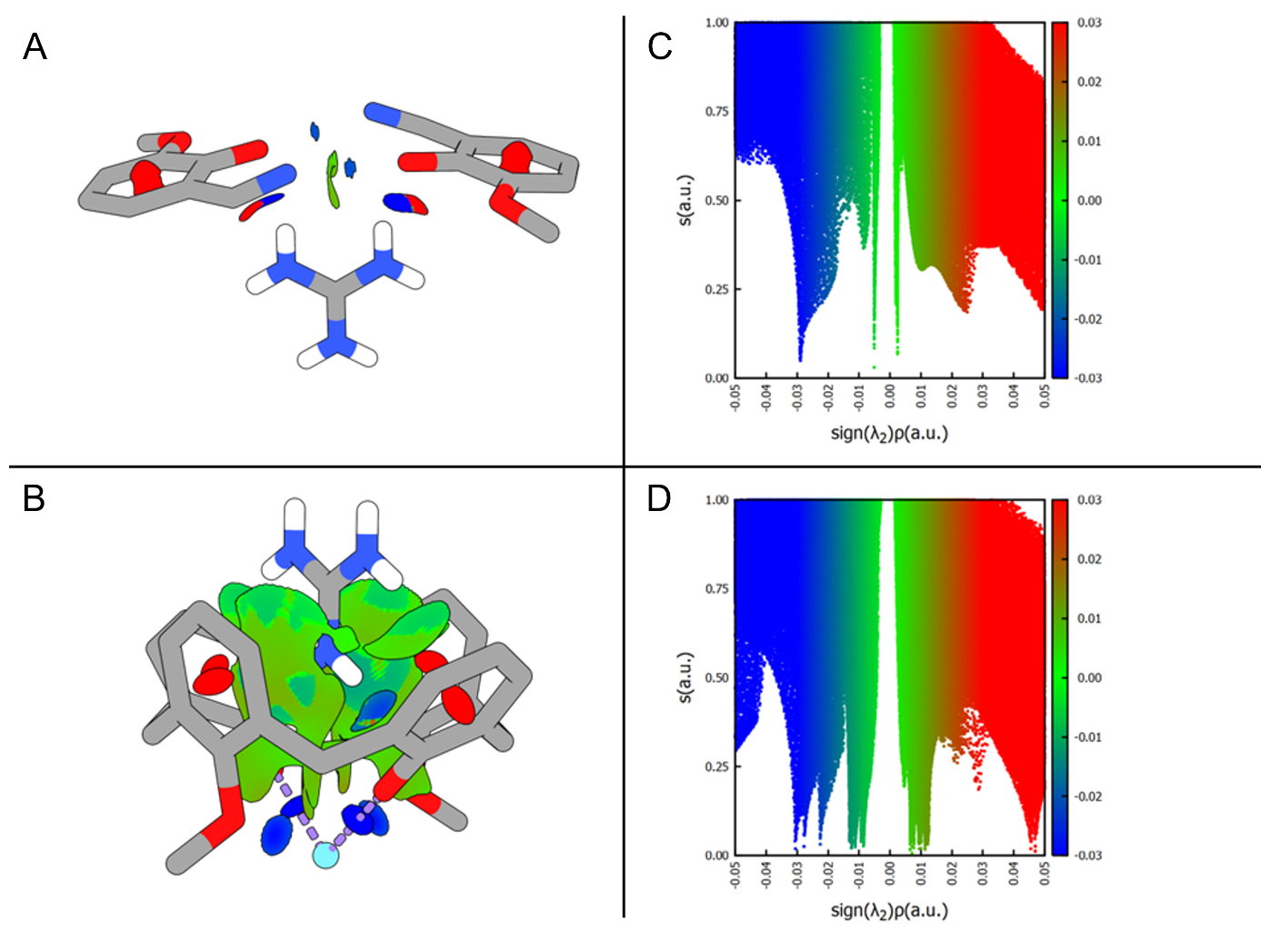


**Figure S6.** A and B: AIM/NCI plots for **2** highlighting the intermolecular NH^…^O (A) and intramolecular (intra-cavity) NH^…^O, NH^…^ π ^(phenolate ring)^, and cation^…^ π ^(phenolether rings)^ interactions (B). C and D: The corresponding RDG scatter diagrams for the two sections. Gradient isosurfaces at 0.3 a.u.

**5. DFT calculations**


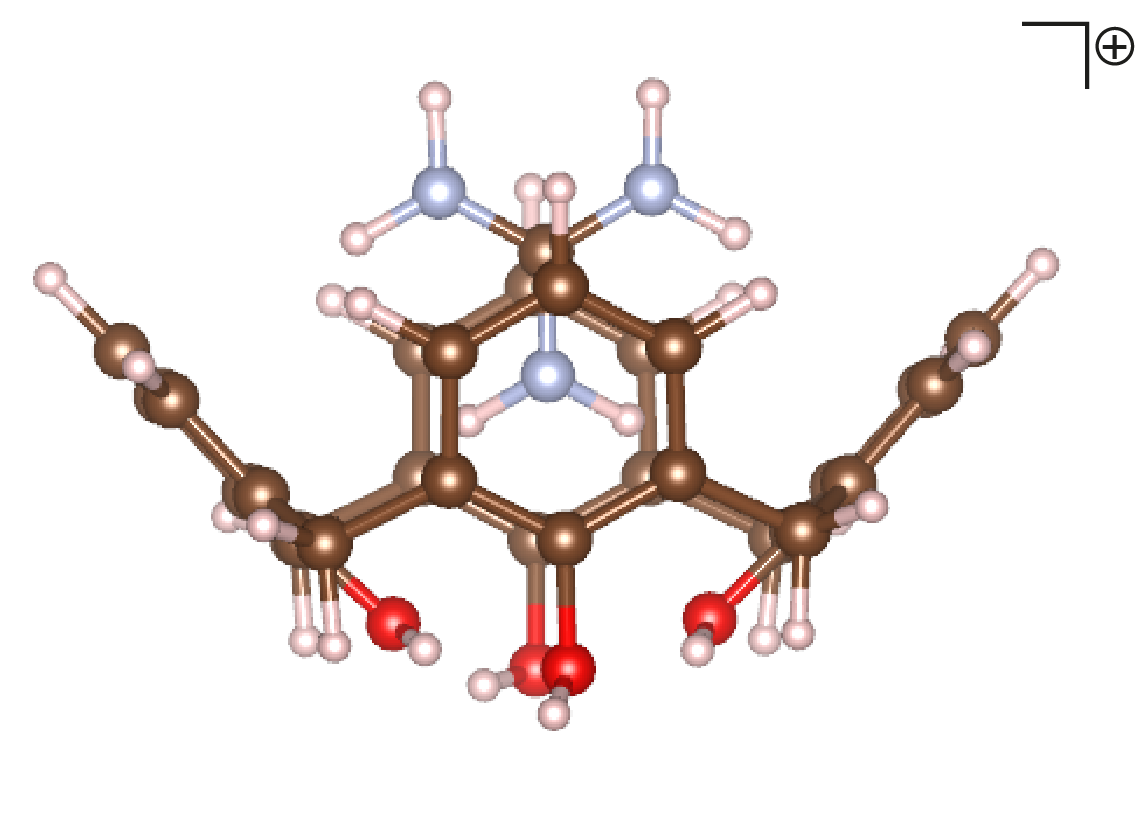


**Figure S7**: Simplified model of complex **2** used for the DFT calculations of the NMR parameters of the guanidinium cation atoms.


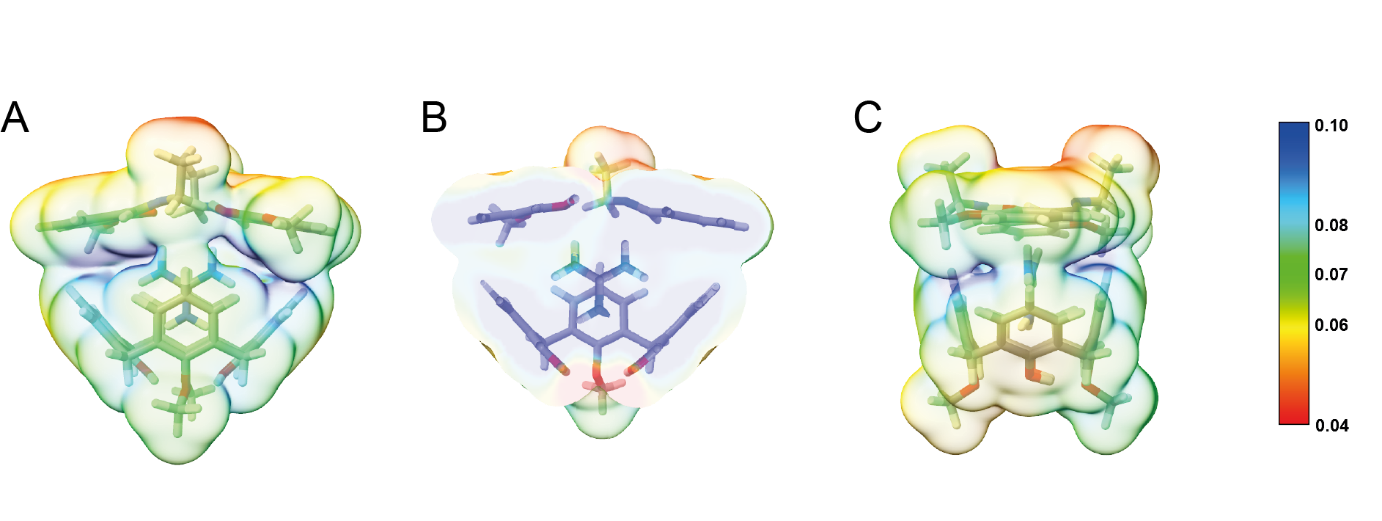


**Figure S8**: The electrostatic potential for the dimeric model of complex **2** as calculated through DFT depicted together with the structure. The color coding of the electrostatic potential given in a.u. follows that of the color bar (right). The H, C, N and O atoms in the structure are coloured white, grey, blue, and red, respectively. The electrostatic potential map is shown for a front (A), a cut-through (B) and a side view of the structure (C). Note that the complex is positively charged (charge +1) and thus generates only a positive electrostatic potential.

**6. Additional NMR data and experimental details**

**6.1. Additional solution state NMR spectra**


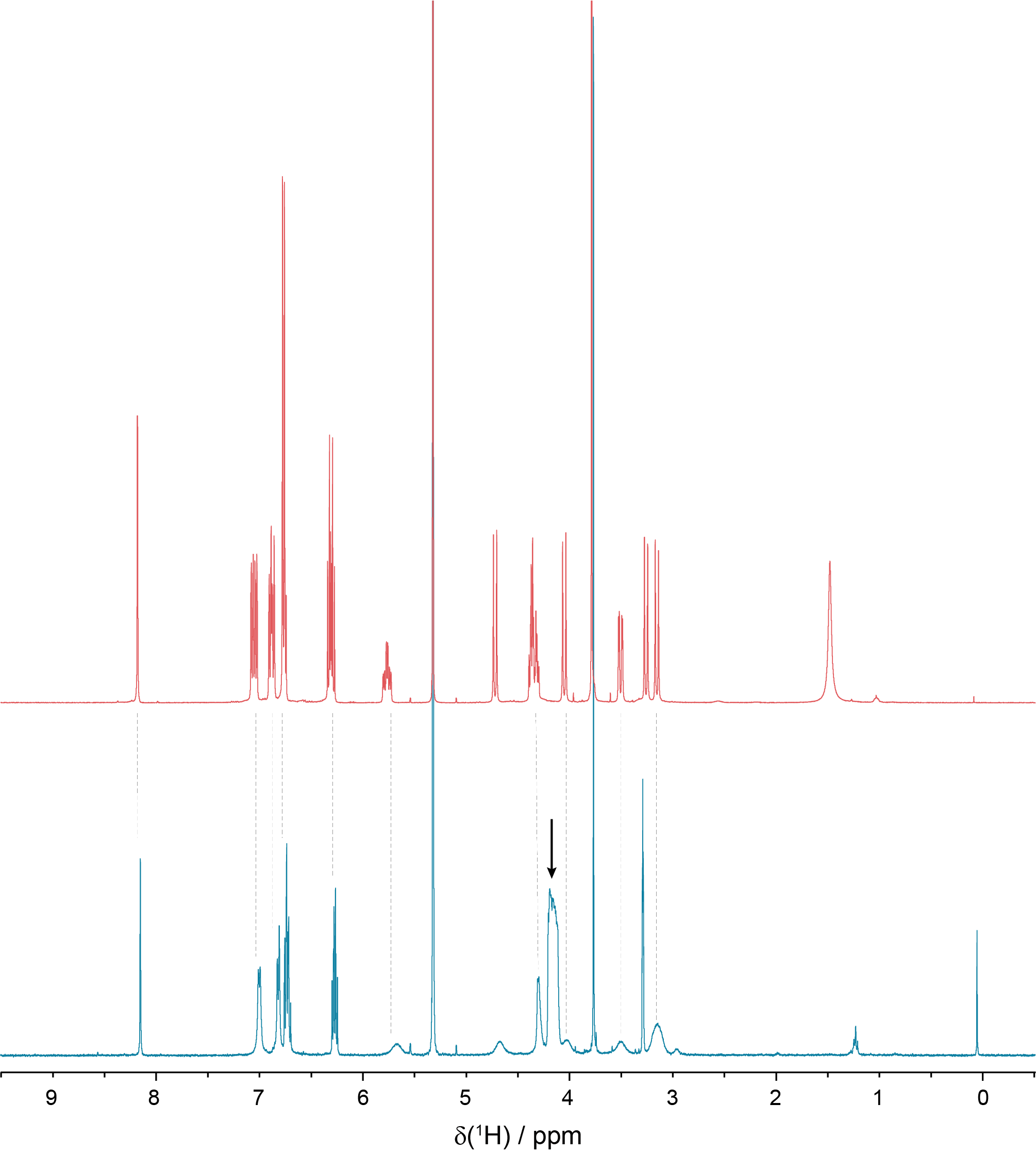


**Figure S9:** Comparison of the solution-state ^1^H NMR spectra of complex **1** (top, red) and the reaction mixture from the sample preparation of complex **2** (bottom, blue). The dashed lines serve as guide to the eye for similar resonances between the two spectra. The arrow marks a broad resonance at 4.16 ppm that is only found in the reaction mixture and is thus assigned to the protons of the trapped guanidinium cation. The chemical-shift value of these protons is strongly influenced by the ring current effect of the aryl rings from the complex leading to a shielding effect in comparison to a “free” guanidinium ion (δ = 7.07 ppm)^[42]^. Both spectra are recorded at room temperature at a 9.4 T spectrometer and in CD_2_Cl_2_/CD_3_OD as solvent.

**6.2. Spectra of natural abundance complex 2 (solid-state NMR)**

**
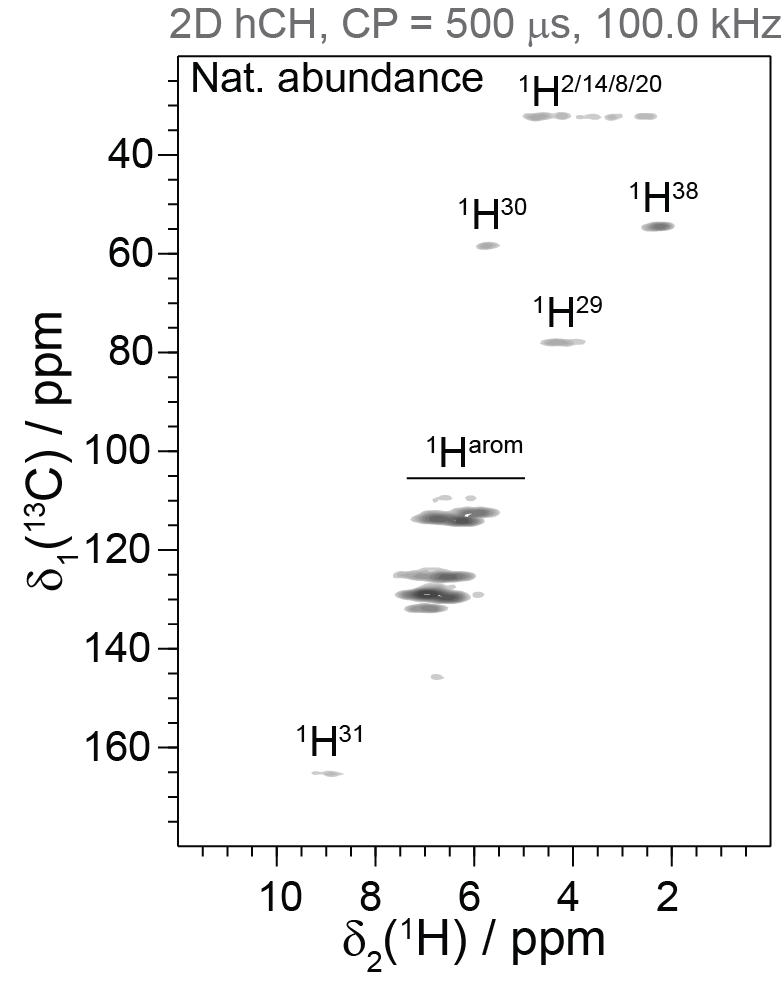
**

**Figure S10:** 2D hCH spectrum showing proton and carbon correlations of complex **2**. The distinguishable spectral regions are marked. The spectrum was recorded with a CP contact time of 500 μs, at a MAS frequency of 100.0 kHz and magnetic-field strength of 16.4 T.

**
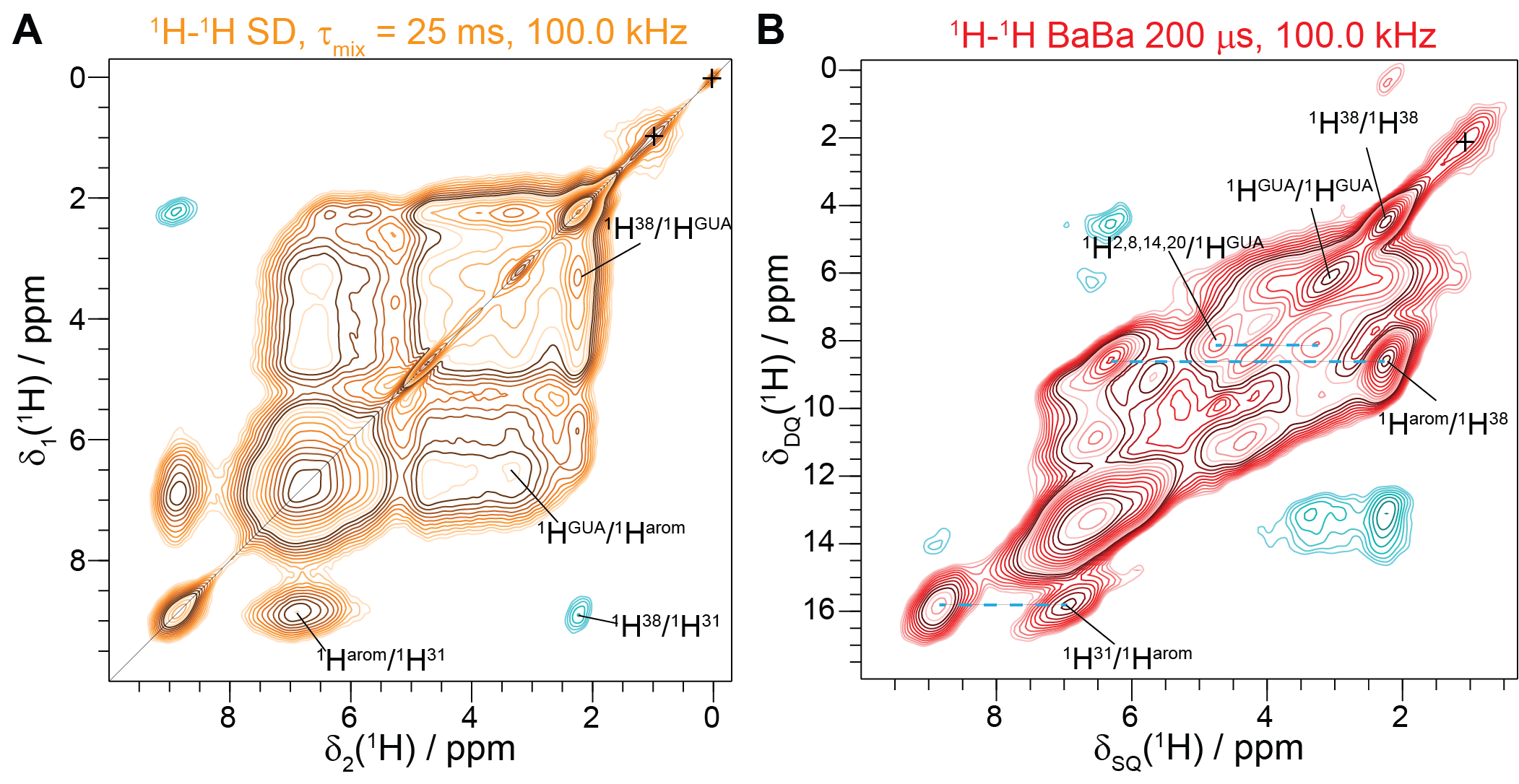
**

**Figure S11:** A 2D ^1^H-^1^H spin diffusion spectrum of complex **2** recorded with a mixing time of 25 ms. Negative cross-peaks arise from higher-order coherent contributions as reported in ^[43]^. B 2D ^1^H-^1^H DQ/SQ spectrum of **2** using BaBa recoupling. Peaks with negative amplitude (cyan contour levels) could in principle originate from three-spin relayed polarization transfer as reported for through-bond HOMCOR experiments^[44]^. Identified correlations are labelled in both cases and minor impurities are marked with a + symbol. Both spectra were recorded with 100.0 kHz MAS and at 16.4 T.

**6.3. Calculation of Gibbs free enthalpy**

The calculation of the Gibbs free enthalpy (*ΔG^‡^*) is performed as described in ref. ^[45]^. First, the coalescence temperature *T_C_* is estimated to 215 ± 15 K from the temperature series of the ^15^N MAS-NMR spectra of **2**-^13^C,^15^N (see Figure 3). Additionally, from the spectrum at the lowest temperature (100 K) the frequency difference of the resonances *Δν =* 372 ± 10 Hz can be determined. With these values the corresponding rate constant *k_ex_* is calculated:

$k_{ex,c}=\frac{\pi\Delta\nu}{\sqrt{2}} \Rightarrow k_{ex,c}=826\pm22 s^{-1}$

Now the Eyring equation is employed and solved towards the Gibbs free enthalpy *ΔG^‡^*:

$$k_{ex}=x\cdot\frac{k_{B}T}{h}\cdotⅇ^{\frac{-\Delta G^{\ddagger}}{RT}} \Longrightarrow\Delta G_{c}^{\ddagger}=-\left( \left( \ln\left( k_{c} \right)- \ln\left( \frac{k_{B} T_{c}}{h} \right) \right) \cdot(RT_{c}) \right)$$

Here *k_B_* refers to the Boltzmann constant, *h* is the Planck constant, *R* is the gas constant, and the transmission coefficient *x* was set to 1. The pair *k_C_* and *T_C_* is plugged into the equation and *ΔG^‡^* is derived. With the given values it is $\Delta G_{c}^{\ddagger}$ *=* 40 ± 3 kJ/mol.

**6.4.** **Nuclear Overhauser Effect: exemplary spectra (solid-state NMR)**


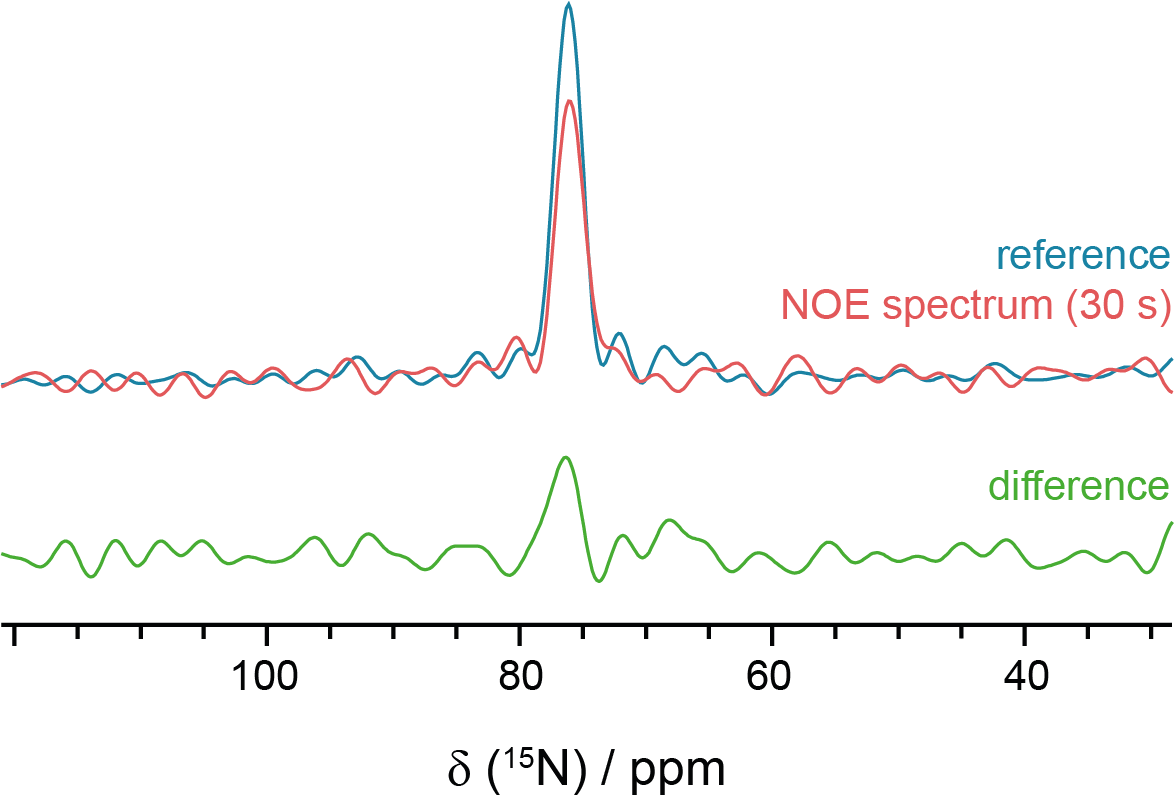


**Figure S12**: Exemplary ^15^N spectra of **2**-^13^C,^15^N for the ^15^N{^1^H} heteronuclear Nuclear Overhauser Effect. The NOE spectrum is recorded with 30 s of proton saturation pulses (red); the reference spectrum is recorded analogous but without the ^1^H saturation (blue). The difference of the two spectra is shown below (green). All spectra were recorded with 60.0 kHz MAS and at 16.4 T.

**6.5. ^13^C-^15^N REDOR experiment to probe dynamics (solid-state NMR)**

The guanidinium cation dynamics was characterized by a ^13^C-^15^N REDOR experiment. From this experiment, the heteronuclear dipolar-coupling constant (and thus internuclear distances) under MAS can be derived^[46-47]^. In case of molecular motion, the REDOR curves are affected by a corresponding modulation of the heteronuclear dipolar-coupling constant^[48-49]^. Figure S11A shows the experimental ^13^C-^15^N REDOR curve for complex **2-**^13^C,^15^N. The experimental data show a significant deviation already in the initial build-up from the rigid limit case represented by a numerical simulation using the SIMPSON program package^[50]^ of the four-spin system for the guanidinium cation based on the distances and orientations from the single-crystal structure. In case of an infinite fast C_3_-rotation, the system can be described in the initial region of the REDOR curve in first approximation by a two-spin system with an effective dipolar coupling constant that is scaled by a factor of -0.5 (given from the value of the second-order Legendre polynomial for an opening angle of 90°)^[48]^. And indeed, the comparison of the experimental REDOR curve with the simulated one based on a two-spin system with an effective dipolar coupling constant of 1196 Hz (fast motional regime) agrees reasonably well for the initial build-up phase (Figure S11A) for ΔS/S_0_ < 0.2^[51]^. Additional simulations were performed with GAMMA^[52]^ taking explicitly the molecular motion (three-site jump model) into account (Figure S11B). Similar simulations have been implemented previously for methyl group rotations, see for instance ^[48]^. The experimental curve follows the simulated one in the fast motional regime (exchange rate *k* = 1 ∙ 10^6^ s^-1^, corresponding to a correlation time *τ_C_* of 333 ns) but is slightly stretched along the dephasing time axis. The origin of this observation remains currently unclear.

*Experimental details REDOR experiment*

The REDOR experiments were performed as reported in ^[47]^ and a control experiment without dipolar recoupling was recorded for each data point to detect a normalized signal. The temperature control was set to a nominal value of 270 K. The geometry independent initial build-up of the REDOR curve^[51]^ was compared to numerically simulated REDOR curves from the software package SIMPSON (Version 5.0)^[50]^ as well as simulation from GAMMA^[52]^ showing the full REDOR curve in dependence of the correlation time of the motion as described in ^[48]^.


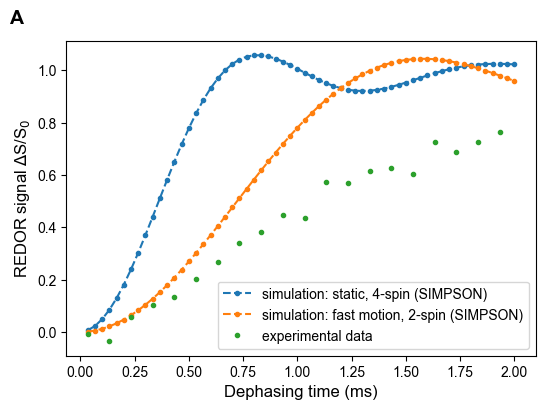


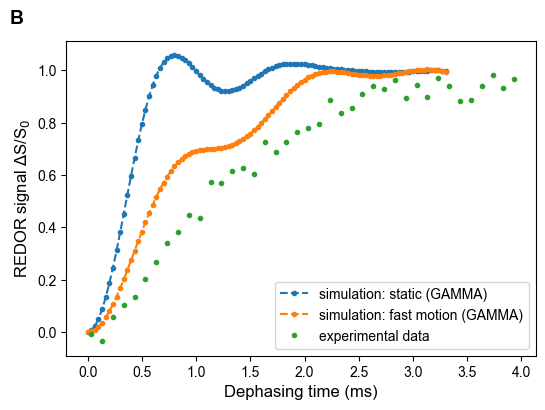


**Figure S13**: A: Experimental ^13^C-^15^N REDOR data (green dots) and SIMPSON simulations^[50]^ for the 4-spin static case with three carbon-nitrogen dipolar couplings of 1305 Hz, 1417 Hz and 1417 Hz (blue) and for the fast motion limit with a 2-spin system and an effective dipolar coupling of 1196 Hz (orange). B: Experimental ^13^C-^15^N REDOR data (green dots) and GAMMA simulations for a static case (blue) and for the fast motion case assuming an exchange rate of *k* = 1 ∙ 10^6^ s, with corresponds to a correlation time *τ_C_* of 333 ns (orange).

**6.6. Guanidinium ^13^C chemical-shift anisotropy (solid-state NMR)**


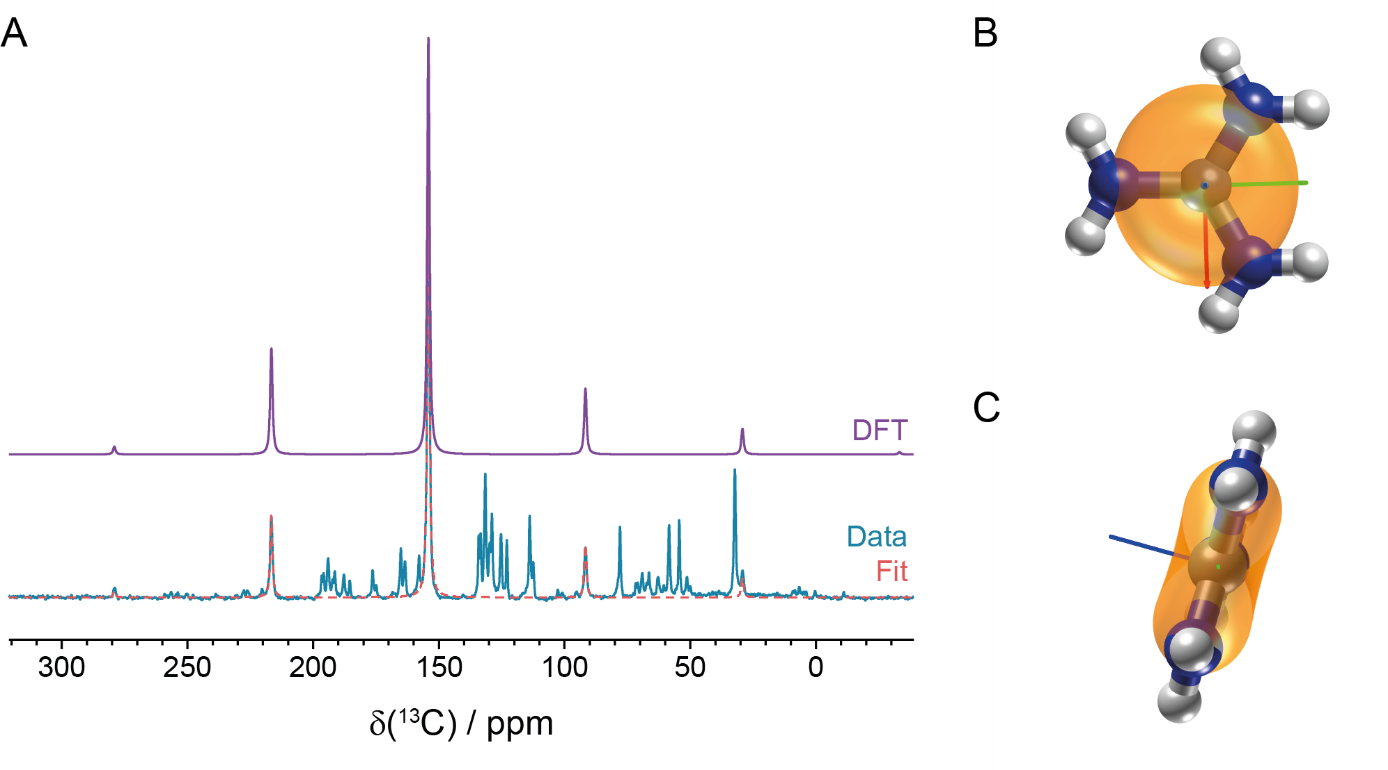


**Figure S14**: Determining the guanidinium ^13^C magnetic-shielding tensor to probe a possible “wobbling” motion. A: ^1^H-^13^C CP solid-state NMR spectrum at 11 kHz MAS frequency at 16.4 T (blue) and line shape fitting for the guanidinium ^13^C resonance (dashed red) with δ_iso_ = 154.3 ppm, Δδ = −91.5 ppm and η = 0.0 for the chemical shift tensor. For comparison the simulated spectrum with the tensor parameters (Δδ = −102.5 ppm and η = 0.16) form DFT is displayed above (purple). B and C: Magnetic shielding tensor as ovaloid and principal axis system (X = red, Y = green, Z = blue) of the guanidinium ion in complex **2** according to DFT calculations (Images generated with TensorView^[53-54]^).

**Table S3**: Guanidinium ^13^C chemical-shift tensor components. All values are expressed according to the Haeberlen Convention with $\Delta\delta=\delta_{zz}^{PAS}- \frac{(\delta_{xx}^{PAS}+\delta_{yy}^{PAS})}{2}$ and $\eta=\frac{\delta_{yy}^{PAS}-\delta_{xx}^{PAS}}{\delta_{zz}^{PAS}-\delta_{iso}}$. The experimental parameters are derived from the fitting of the slow MAS NMR spectrum (Figure S12) and the magnetic-shielding values obtained from DFT calculations were referenced to the chemical shift.

|  | **δ_iso_ [ppm]** | **Δδ [ppm]** | **η** | **δ_xx_ [ppm]** | **δ_yy_ [ppm]** | **δ_zz_ [ppm]** |
| --- | --- | --- | --- | --- | --- | --- |
| **Experimental** | 154.3 | −91.5 | 0.00 | 199.9 | 199.9 | 62.7 |
| **DFT** | 155.8 | −102.5 | 0.16 | 215.2 | 199.0 | 53.3 |

**6.7. Deuterium NMR (solid-state NMR)**

To probe NH_2_ flips, a slow-spinning ^2^H MAS-NMR spectrum on complex **2**-^2^H was recorded as the quadrupolar coupling constant, *C_Q_,* serves as a highly sensitive probe for molecular motion. The line shape analysis reveals a *C_Q_*-value of around 150 kHz with an η_Q_-value of 0.12. For rigid guanidinium cations in chloride, urea and other salts *C_Q_*-values of 200 to 250 kHz were reported with η_Q_-values ranging in-between 0.12-0.15^[55]^. At higher temperatures, axially symmetric quadrupolar-coupling tensors (η_Q_=0) with a reduced *C*_Q_-value of 108-113 kHz have been found, which has been attributed to the rapid C_3_ rotation of the guanidinium cation, although NH_2_-flips could not be ruled out. The comparison of these numbers indicates that the C_3_-rotation is probably slower in our case, which still leads to the retention of a non-axial tensor. However, the individual contributions of C_3_-rotation and/or NH_2_ flips to the observed reduction in *C*_Q_-values cannot be determined unambiguously.


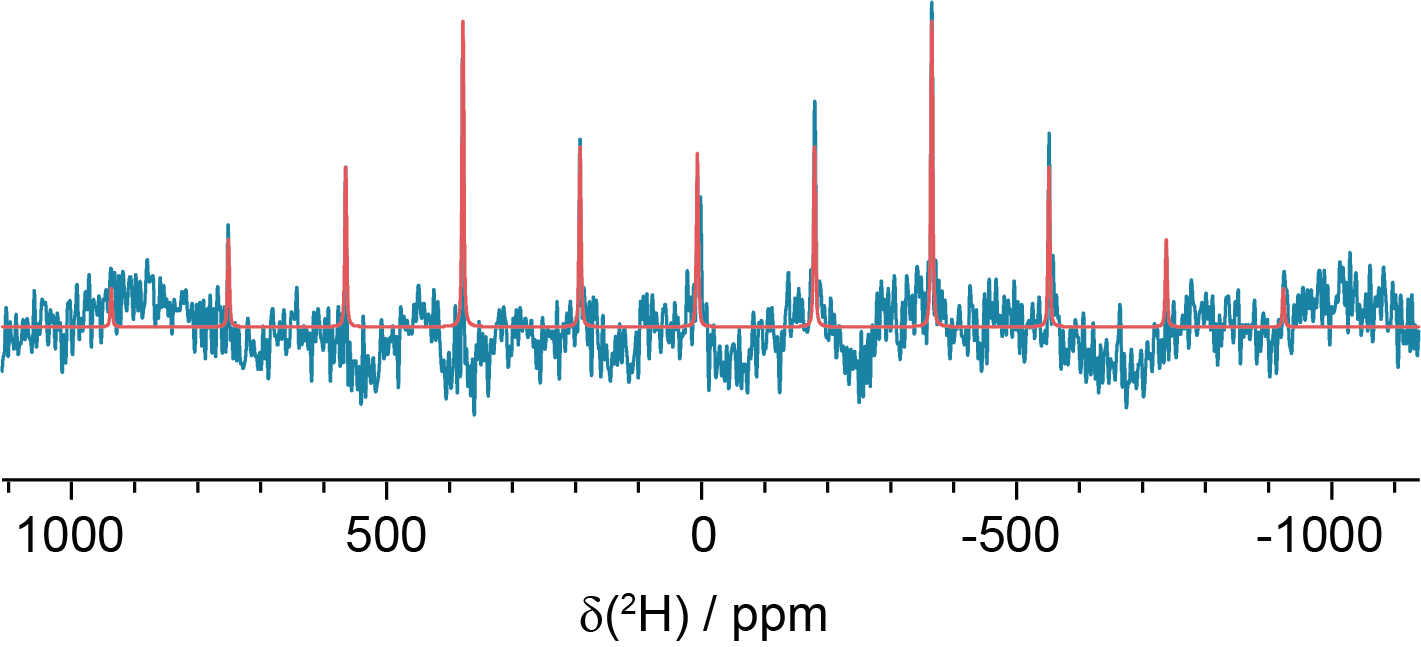


**Figure S15**: ^2^H spectrum of **2**-^2^H at 20 kHz MAS frequency at 16.4 T (blue) and the corresponding line shape fitting (red) with δ_iso_ = 5.9 ppm, C_Q_ = 156 kHz and η_σ_ = 0.12 for the quadrupolar coupling tensor. Note that the full recorded spectrum has a width of 500 kHz, depicted here is only the relevant region of interest.

**6.8. Experimental data (solid-state NMR)**

**Table S4**: Experimental details on solid-state MAS NMR spectra. Raw data on all experiments can be found here: (DOI) 10.22000/ytfddayjzw0nx4p5

| **^1^H Hahn echo** | [(C(NH_2_)_3_)LaL] **(2)** | | | | | | |
| --- | --- | --- | --- | --- | --- | --- | --- |
| *ν_R_* [kHz] | 40 | 50 | 60 | 70 | 80 | 90 | 100 |
| *B_0_* [T] | 16.4 | 16.4 | 16.4 | 16.4 | 16.4 | 16.4 | 16.4 |
| *ν_1_* (^1^H) [kHz] | 150 | 150 | 150 | 150 | 150 | 150 | 150 |
| ^1^H carrier [ppm] | 2.6 | 2.6 | 2.6 | 2.6 | 2.6 | 2.6 | 2.6 |
| Time domain points | 16384 | 16384 | 16384 | 16384 | 16384 | 16384 | 16384 |
| Sweep width [ppm] | 142.8 | 142.8 | 142.8 | 142.8 | 142.8 | 142.8 | 142.8 |
| Acquisition time [ms] | 81.9 | 81.9 | 81.9 | 81.9 | 81.9 | 81.9 | 81.9 |
| Interscan delay [s] | 14.5 | 14.5 | 14.5 | 14.5 | 14.5 | 14.5 | 14.5 |
| Number of Scans | 16 | 16 | 16 | 16 | 16 | 16 | 32 |
| Probe target temperature [K] | 287 | 287 | 287 | 287 | 287 | 287 | 287 |

| **^1^H-^13^C CP** | [(C(NH_2_)_3_)LaL] **(2)** | [(^13^C(^15^NH_2_)_3_)LaL] **(2-**^13^C, ^15^N**)** |
| --- | --- | --- |
| *ν_R_* [kHz] | 60 | 60 |
| *B_0_* [T] | 16.4 | 16.4 |
| **Transfer** | H-C CP | H-C CP |
| *ν_CP_* (^1^H) [kHz] | 100 | 100 |
| *ν_CP_* (^13^C) [kHz] | 38 | 36 |
| Shape | ^1^H Tangent shape | ^1^H Tangent shape |
| CP contact time [ms] | 5 | 3 |
| ^1^H carrier [ppm] | 7.1 | 6.1 |
| ^1^H decoupling power [kHz] | 5 | 5 |
| ^13^C carrier [ppm] | 99 | 155 |
| Time domain points | 8192 | 8192 |
| Sweep width [ppm] | 567.8 | 591.5 |
| Acquisition time [ms] | 41.0 | 39.3 |
| Interscan delay [s] | 14.5 | 14.0 |
| Number of Scans | 8192 | 800 |
| Probe target temperature [K] | 282 | 290 |

| **1D hNH** | [(^13^C(^15^NH_2_)_3_)LaL] **(2-**^13^C, ^15^N**)** |
| --- | --- |
| *ν_R_* [kHz] | 60 |
| *B_0_* [T] | 16.4 |
| **Transfer I** | H-N CP |
| *ν_CP_* (^1^H) [kHz] | 100 |
| *ν_CP_* (^15^N) [kHz] | 51 |
| Shape | ^1^H Tangent shape |
| CP contact time [ms] | 0.15 |
| **Transfer II** | N-H CP |
| *ν_CP_* (^1^H) [kHz] | 100 |
| *ν_CP_* (^15^N) [kHz] | 51 |
| Shape | ^1^H Tangent shape |
| CP contact time [ms] | 0.15 |
| ^1^H carrier [ppm] | 4.7 |
| ^15^N carrier [ppm] | 80.3 |
| ^15^N decoupling power [kHz] | 5 |
| Time domain points | 8192 |
| Sweep width [ppm] | 142.8 |
| Acquisition time [ms] | 41.0 |
| Interscan delay [s] | 14.5 |
| Number of Scans | 2048 |
| Probe target temperature [K] | 270 |
| **^1^H Hahn echo** | [(^13^C(^15^NH_2_)_3_)LaL] **(2-**^13^C, ^15^N**)** |
| *ν_R_* [kHz] | 60 |
| *B_0_* [T] | 16.4 |
| *ν_1_* (^1^H) [kHz] | 100 |
| ^1^H carrier [ppm] | 4.7 |
| Time domain points | 16384 |
| Sweep width [ppm] | 142.8 |
| Acquisition time [ms] | 81.9 |
| Interscan delay [s] | 14.5 |
| Number of Scans | 64 |
| Probe target temperature [K] | 270 |

| **2D hCH** | [(^13^C(^15^NH_2_)_3_)LaL] **(2-**^13^C, ^15^N**)** | [(C(NH_2_)_3_)LaL] **(2)** |
| --- | --- | --- |
| *ν_R_* [kHz] | 60 | 100 |
| *B_0_* [T] | 16.4 | 16.4 |
| **Transfer I** | H-C CP | H-C CP |
| *ν_CP_* (^1^H) [kHz] | 100 | 110 |
| *ν_CP_* (^13^C) [kHz] | 33 | 20 |
| Shape | ^1^H Tangent shape | ^1^H Tangent shape |
| CP contact time [ms] | 1 | 0.5 |
| **Transfer II** | C-H CP | H-C CP |
| *ν_CP_* (^1^H) [kHz] | 100 | 110 |
| *ν_CP_* (^13^C) [kHz] | 33 | 20 |
| Shape | ^1^H Tangent shape | ^1^H Tangent shape |
| CP contact time [ms] | 1 | 0.5 |
| ^1^H carrier [ppm] | 4.4 | 0.6 |
| ^13^C carrier [ppm] | 95.0 | 99.5 |
| ^13^C decoupling power [kHz] | 5 | 5 |
| Time domain points F1 | 256 | 432 |
| Sweep width F1 [ppm] | 200 | 200 |
| Acquisition time F1 [ms] | 36.3 | 6.1 |
| Time domain points F2 | 8192 | 8192 |
| Sweep width F2 [ppm] | 142.8 | 142.8 |
| Acquisition time F2 [ms] | 41.0 |  |
| Interscan delay [s] | 14 | 41.0 |
| Number of Scans | 32 | 32 |
| Probe target temperature [K] | 282 | 289 |

| **2D ^1^H-^1^H Spin Diffusion** | [(C(NH_2_)_3_)LaL] **(2)** | |
| --- | --- | --- |
| *ν_R_* [kHz] | 100 | 100 |
| *B_0_* [T] | 16.4 | 16.4 |
| **Transfer** | Spin Diffusion | BaBa recoupling |
| Mixing time [ms] | 25 | 200 |
| ^1^H carrier [ppm] | 6.1 | 6.1 |
| Time domain points F1 | 512 | 512 |
| Sweep width F1 [ppm] | 42.8 | 142.8 |
| Acquisition time F1 [ms] | 8.5 | 2.5 |
| Time domain points F2 | 16384 | 16384 |
| Sweep width F2 [ppm] | 142.8 | 142.8 |
| Acquisition time F2 [ms] | 81.9 | 81.9 |
| Interscan delay [s] | 14.5 | 14.5 |
| Number of Scans | 16 | 16 |
| Probe target temperature [K] | 289 | 289 |

| **^1^H-^15^N CP (low temperature)** | [(^13^C(^15^NH_2_)_3_)LaL] **(2-**^13^C, ^15^N**)** |
| --- | --- |
| *ν_R_* [kHz] | 18 |
| *B_0_* [T] | 9.4 |
| **Transfer** | H-N CP |
| *ν_CP_* (^1^H) [kHz] | 110 |
| *ν_CP_* (^15^N) [kHz] | 49 |
| Shape | ^1^H ramp |
| CP contact time [ms] | 0.5 |
| ^1^H carrier [ppm] | 4.6 |
| ^1^H decoupling power [kHz] | 139 |
| ^15^N carrier [ppm] | 36.3 |
| Time domain points | 2048 |
| Sweep width [ppm] | 1027 |
| Acquisition time [ms] | 24.6 |
| Interscan delay [s] | 3 |
| Number of Scans | 64 |
| Probe target temperature [K] | 95-243 |

| **^15^N NOE** | [(^13^C(^15^NH_2_)_3_)LaL] **(2-**^13^C, ^15^N**)** |
| --- | --- |
| *ν_R_* [kHz] | 60 |
| *B_0_* [T] | 16.4 |
| *ν_1_* (^15^N) [kHz] | 83 |
| ^1^H carrier [ppm] | 5.8 |
| ^1^H decoupling power [kHz] | 5 |
| ^15^N carrier [ppm] | 72.3 |
| Time domain points | 4096 |
| Sweep width [ppm] | 1409 |
| Acquisition time [ms] | 20.5 |
| Interscan delay [s] | 52 |
| Number of Scans | 128 |
| Probe target temperature [K] | 270 |

| **^1^H-^13^C CP (slow MAS)** | [(^13^C(^15^NH_2_)_3_)LaL] **(2-**^13^C, ^15^N**)** |
| --- | --- |
| *ν_R_* [kHz] | 11 |
| *B_0_* [T] | 16.4 |
| **Transfer** | H-C CP |
| *ν_CP_* (^1^H) [kHz] | 50 |
| *ν_CP_* (^13^C) [kHz] | 41 |
| Shape | ^1^H Tangent shape |
| CP contact time [ms] | 3 |
| ^1^H carrier [ppm] | 5.4 |
| ^1^H decoupling power [kHz] | 90 |
| ^13^C carrier [ppm] | 154.5 |
| Time domain points | 4096 |
| Sweep width [ ppm] | 591 |
| Acquisition time [ms] | 19.7 |
| Interscan delay [s] | 14 |
| Number of Scans | 5376 |
| Probe target temperature [K] | 280 |

| **^2^H (slow MAS)** | [(C(N^2^H_2_)_3_)LaL] **(2-**^2^H**)** |
| --- | --- |
| *ν_R_* [kHz] | 20 |
| *B_0_* [T] | 16.4 |
| *ν_1_* (^2^H) [kHz] | 76 |
| ^1^H carrier [ppm] | 12.2 |
| ^1^H decoupling power [kHz] | 90 |
| ^2^H carrier [ppm] | 10.6 |
| Time domain points | 4096 |
| Sweep width [ppm] | 4651.0 |
| Acquisition time [ms] | 4.1 |
| Interscan delay [s] | 1.5 |
| Number of Scans | 77824 |
| Probe target temperature [K] | 295 |

| **^13^C-^15^N REDOR** | [(C(N^2^H_2_)_3_)LaL] **(2-**^2^H**)** |
| --- | --- |
| *ν_R_* [kHz] | 60 |
| *B_0_* [T] | 16.4 |
| **Transfer** | H-C CP |
| *ν_CP_* (^1^H) [kHz] | 90 |
| *ν_CP_* (^13^C) [kHz] | 42 |
| Shape | ^1^H Tangent shape |
| CP contact time [ms] | 1 |
| ^1^H carrier [ppm] | 5.4 |
| ^1^H decoupling power [kHz] | 5 |
| ^13^C carrier [ppm] | 156.1 |
| ^13^C recoupling pulse [kHz] | 100 |
| ^15^N carrier [ppm] | 78.7 |
| ^15^N recoupling pulse [kHz] | 83 |
| Time domain points | 3072 |
| Sweep width [ppm] | 591 |
| Acquisition time [ms] | 14.7 |
| Interscan delay [s] | 16 |
| Number of Scans | 16 |
| Probe target temperature [K] | 270 |

| **Saturation recovery (^13^C, ^15^N)** | [(^13^C(^15^NH_2_)_3_)LaL] **(2-*^13^C***, ^15^N**)** | [(^13^C(^15^NH_2_)_3_)LaL] **(2-**^13^C, ***^15^N*)** |
| --- | --- | --- |
| *ν_R_* [kHz] | 60 | 60 |
| *B_0_* [T] | 16.4 | 16.4 |
| *ν_1_* (^13^C) / *ν_1_* (^15^N) [kHz] | 100 | 83 |
| ^13^C / ^15^N carrier [ppm] | 166.0 | 78.7 |
| ^1^H carrier [ppm] | 6.1 | 6.1 |
| ^1^H decoupling power [kHz] | 5 | 5 |
| Time domain points | 4096 | 4096 |
| Sweep width [ ppm] | 591 | 1468 |
| Acquisition time [ms] | 19.7 | 19.7 |
| Interscan delay [s] | 0.5 | 1 |
| Number of Scans | 16 | 32 |
| Probe target temperature [K] | 260 / 270 / 280 / 290 | 260 / 270 / 280 / 290 |

**7. Molecular Dynamics Simulation**

**
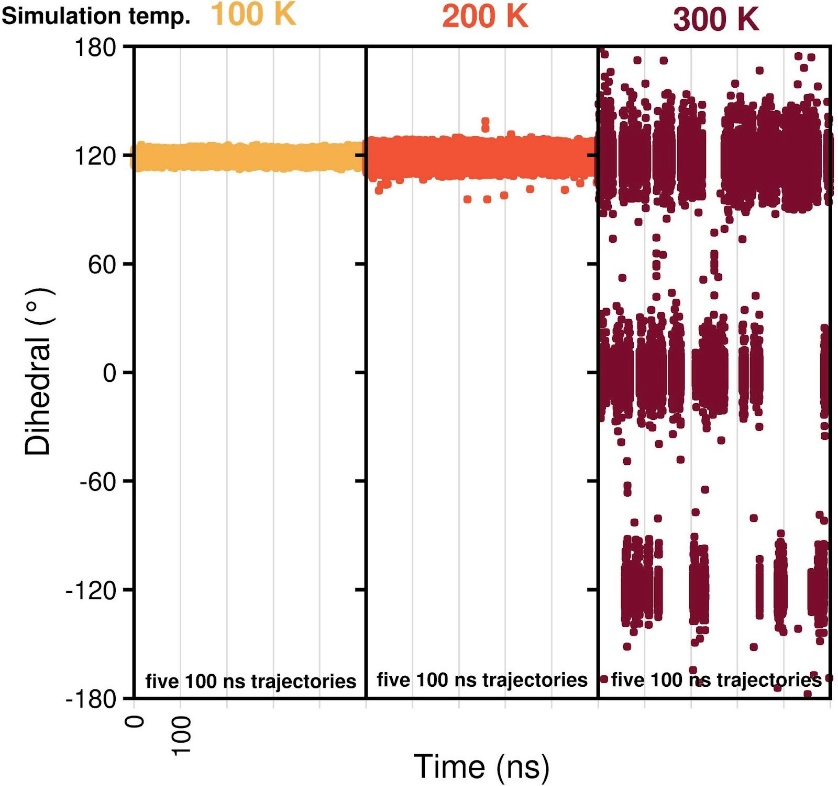
**

**Figure S16**: The time trace of the dihedral angle corresponding to the C_3_ rotation of the guanidinium rotor at three different temperatures, obtained from a total of 500 ns long MD simulations at each temperature.

**Video S1**: Video of the MD simulation available as separate file.

**8. Supplementary references**

[1] S. Ullmann, R. Schnorr, C. Laube, B. Abel, B. Kersting, *Dalton Trans.* **2018**, *47*, 5801-5811.

[2] OriginPro (Version 2019), OriginLab Corporation, Northampton, MA, USA., **2019**.

[3] E. R. Johnson, S. Keinan, P. Mori-Sanchez, J. Contreras-Garcia, A. J. Cohen, W. Yang, *J. Am. Chem. Soc.* **2010**, *132*, 6498-6506.

[4] J. Contreras-Garcia, E. R. Johnson, S. Keinan, R. Chaudret, J. P. Piquemal, D. N. Beratan, W. Yang, *J. Chem. Theory Comput.* **2011**, *7*, 625-632.

[5] T. Williams, C. Kelley, gnuplot 5.4: An Interactive Plotting Program, **2023**.

[6] E. C. Meng, T. D. Goddard, E. F. Pettersen, G. S. Couch, Z. J. Pearson, J. H. Morris, T. E. Ferrin, *Protein Sci.* **2023**, *32*, e4792.

[7] E. F. Pettersen, T. D. Goddard, C. C. Huang, E. C. Meng, G. S. Couch, T. I. Croll, J. H. Morris, T. E. Ferrin, *Protein Sci.* **2021**, *30*, 70-82.

[8] G. M. Sheldrick, *Acta Crystallogr. A* **2015**, *71*, 3-8.

[9] O. V. Dolomanov, L. J. Bourhis, R. J. Gildea, J. A. K. Howard, H. Puschmann, *J. Appl. Crystallogr.* **2009**, *42*, 339-341.

[10] F. Neese, *Wiley Interdiscip. Rev. Comput. Mol. Sci.* **2022**, *12*.

[11] F. Neese, *Wiley Interdiscip. Rev. Comput. Mol. Sci.* **2025**, *15*.

[12] S. Grimme, A. Hansen, S. Ehlert, J. M. Mewes, *J. Chem. Phys.* **2021**, *154*.

[13] F. Weigend, *Phys. Chem. Chem. Phys.* **2006**, *8*, 1057-1065.

[14] E. Caldeweyher, C. Bannwarth, S. Grimme, *J. Chem. Phys.* **2017**, *147*, 034112.

[15] H. Kruse, S. Grimme, *J. Chem. Phys.* **2012**, *136*, 154101.

[16] C. Adamo, V. Barone, *J. Chem. Phys.* **1999**, *110*, 6158-6170.

[17] F. Jensen, *J. Chem. Theory Comput.* **2015**, *11*, 132-138.

[18] G. L. Stoychev, A. A. Auer, R. Izsak, F. Neese, *J. Chem. Theory Comput.* **2018**, *14*, 619-637.

[19] S. F. Boys, F. Bernardi, *Mol. Phys.* **1970**, *19*, 553–566.

[20] M. J. Abraham, T. Murtola, R. Schulz, S. Páll, J. C. Smith, B. Hess, E. Lindahl, *SoftwareX* **2015**, *1–2*, 19–25.

[21] J. Wang, R. M. Wolf, J. W. Caldwell, P. A. Kollman, D. A. Case, *J. Comput. Chem.* **2004**, *25*, 1157-1174.

[22] P. Li, L. F. Song, K. M. Merz, Jr., *J. Phys. Chem. B* **2015**, *119*, 883-895.

[23] B. Hess, C. Kutzner, D. van der Spoel, E. Lindahl, *J. Chem. Theory Comput.* **2008**, *4*, 435-447.

[24] G. Bussi, D. Donadio, M. Parrinello, *J. Chem. Phys.* **2007**, *126*.

[25] T. Darden, D. York, L. Pedersen, *J. Chem. Phys.* **1993**, *98*, 10089-10092.

[26] R. K. Harris, E. D. Becker, S. M. C. De Menezes, P. Granger, R. E. Hoffman, K. W. Zilm, *Magn. Reson. Chem.* **2008**, *46*, 582–598.

[27] S. Hediger, B. H. Meier, N. D. Kurur, G. Bodenhausen, R. R. Ernst, *Chem. Phys. Lett.* **1994**, *223*, 283-288.

[28] S. Hediger, B. H. Meier, R. R. Ernst, *Chem. Phys. Lett.* **1995**, *240*, 449-456.

[29] A. J. Shaka, J. Keeler, *Prog. Nucl. Magn. Reson. Spectrosc.* **1987**, *19*, 47-129.

[30] K. R. Thurber, R. Tycko, *J. Magn. Reson.* **2009**, *196*, 84-87.

[31] C. Ye, R. Fu, J. Hu, L. Hou, S. Ding, *Magn. Reson. Chem.* **1993**, *31*, 699–704.

[32] J. S. Higgins, A. H. Hodgson, R. V. Law, *J. Mol. Struct.* **2002**, *602*, 505-510.

[33] S. G. J. van Meerten, W. M. J. Franssen, A. P. M. Kentgens, *J. Magn. Reson.* **2019**, *301*, 56-66.

[34] D. Neuhaus, M. P. Williamson, *The nuclear Overhauser effect in structural and conformational analysis*, VCH, **1992**.

[35] I. M. Walker, P. J. Mccarthy, *Inorg. Chem.* **1992**, *31*, 4122-4127.

[36] E. Bartalucci, A. A. Malar, A. Mehnert, J. B. Kleine Buning, L. Gunzel, M. Icker, M. Borner, C. Wiebeler, B. H. Meier, S. Grimme, B. Kersting, T. Wiegand, *Angew. Chem. Int. Ed. Engl.* **2023**, *62*, e202217725.

[37] L. J. Farrugia, *J. Appl. Crystallogr.* **2012**, *45*, 849–854.

[38] POV-Ray, version 3.6, Persistence of Vision Pty. Ltd., 2004.

[39] M. Llunell, D. Casanova, J. Cirera, P. Alemany, S. Alvarez, SHAPE, version 2.1, Universitat de Barcelona, Barcelona, Spain, **2013**.

[40] R. F. W. Bader, *Atoms in Molecules: A Quantum Theory*, Oxford University PressOxford, **1990**.

[41] R. F. W. Bader, *J. Phys. Chem. A* **1998**, *102*, 7314-7323.

[42] G. A. Olah, A. Burrichter, G. Rasul, M. Hachoumy, G. K. S. Prakash, *J. Am. Chem. Soc.* **1997**, *119*, 12929-12933.

[43] V. Agarwal, *J. Magn. Reson.* **2020**, *311*, 106661-106661.

[44] Q. Wang, B. Hu, O. Lafon, J. Trebosc, F. Deng, J. P. Amoureux, *J. Magn. Reson.* **2010**, *203*, 113-128.

[45] H. Friebolin, *Ein- und zweidimensionale NMR-Spektroskopie*, Wiley-VCH, **2011**.

[46] P. Schanda, M. Ernst, *Prog. Nucl. Magn. Reson. Spectrosc.* **2016**, *96*, 1-46.

[47] T. Gullion, J. Schaefer, *J. Magn. Reson.* **1989**, *81*, 196-200.

[48] K. Aebischer, L. M. Becker, P. Schanda, M. Ernst, *Magn. Reson.* **2024**, *5*, 69-86.

[49] M. R. Hansen, R. Graf, H. W. Spiess, *Acc. Chem. Res.* **2013**, *46*, 1996-2007.

[50] M. Bak, J. T. Rasmussen, N. C. Nielsen, *J. Magn. Reson.* **2000**, *147*, 296-330.

[51] M. Bertmer, H. Eckert, *Solid State Nucl. Magn. Reson.* **1999**, *15*, 139-152.

[52] S. A. Smith, T. O. Levante, B. H. Meier, R. R. Ernst, *J. Magn. Reson., Ser A* **1994**, *106*, 75-105.

[53] R. P. Young, C. R. Lewis, C. Yang, L. Wang, J. K. Harper, L. J. Mueller, *Magn. Reson. Chem.* **2019**, *57*, 211-223.

[54] L. Svenningsson, L. J. Mueller, *Solid State Nucl. Magn. Reson.* **2023**, *123*, 101849.

[55] C. I. Ratcliffe, *Can. J. Chem.* **1985**, *63*, 1239-1244.
